# YiaC and CobB regulate lysine lactylation in *Escherichia coli*

**DOI:** 10.1038/s41467-022-34399-y

**Published:** 2022-11-04

**Authors:** Hanyang Dong, Jianji Zhang, Hui Zhang, Yue Han, Congcong Lu, Chen Chen, Xiaoxia Tan, Siyu Wang, Xue Bai, Guijin Zhai, Shanshan Tian, Tao Zhang, Zhongyi Cheng, Enmin Li, Liyan Xu, Kai Zhang

**Affiliations:** 1 The Province and Ministry Co-sponsored Collaborative Innovation Center for Medical Epigenetics, Key Laboratory of Immune Microenvironment and Disease (Ministry of Education), Tianjin Key Laboratory of Medical Epigenetics, Department of Biochemistry and Molecular Biology, School of Basic Medical Sciences, Tianjin Medical University, 300070 Tianjin, China; 2 Guangdong Provincial Key Laboratory of Infectious Diseases and Molecular Immunopathology, Institute of Oncologic Pathology, Shantou University Medical College, 515041 Shantou, Guangdong China; 3 College of Life Sciences, Nankai University, 300071 Tianjin, China; 4 Jingjie PTM Biolab (Hangzhou) Co. Ltd, Hangzhou, 310018 Zhejiang, China; 5 The Key Laboratory of Molecular Biology for High Cancer Incidence Coastal Chaoshan Area, Department of Biochemistry and Molecular Biology, Shantou University Medical College, 515041 Shantou, Guangdong China; 6 Tianjin Key Laboratory of Retinal Functions and Diseases, Eye Institute and School of Optometry, Tianjin Medical University Eye Hospital, Tianjin Medical University, 300070 Tianjin, China; 7 Tianjin Key Laboratory of Digestive Diseases, Department of Gastroenterology and Hepatology, Medical University General Hospital, Tianjin Medical University, 300070 Tianjin, China

**Keywords:** Chemical modification, Proteomics, Bacteria, Post-translational modifications

## Abstract

Lysine lactylation (Kla) has recently been reported to participate in regulating transcription in human cells. However, the characterization, regulatory mechanism and functional consequence of Kla in prokaryotes remain unclear. Here, we report that YiaC functions as a lysine lactylase and that CobB serves as a lysine delactylase in the regulation of metabolism. We demonstrate that YiaC catalyzes the addition of Kla, while CobB erases this PTM both in vitro and intracellularly. Moreover, we show that YdiF can catalyze the formation of a lactyl-coenzyme A, which donates lactyl group for Kla. Quantitative proteomic analysis further reveals 446 endogenous Kla sites targeted by CobB and 79 candidates targeted by YiaC in *Escherichia coli* (*E. coli*). Furthermore, we present that Kla can influence the functions of metabolic enzymes. Interestingly, we demonstrate that CobB can specifically modulate the activity of PykF by regulating K382la, promoting glycolysis and bacterial growth. Our study identifies the regulatory enzymes and functional network of Kla and reveals a Kla-mediated molecular mechanism catalyzed by CobB for glycolysis regulation in *E. coli*.

## Abstract (web summary)

The characterization of lysine lactylation (Kla) in prokaryotes remains unclear. Here, the authors identify the regulatory enzymes (YiaC as a lactylase and CobB as a delactylase) and functional network of Kla and reveal a Kla-mediated molecular mechanism for glycolysis regulation in Escherichia coli.

## Introduction

Posttranslational modification (PTM) is a key element in the regulation of protein functions and plays a vital role in modulating diverse cellular functions and disease progression in eukaryotes^1,2^. Moreover, increasing evidence indicates that PTMs also perform an important function in prokaryotes. For example, lysine acetylation (Kac) regulates bacterial virulence^3^, chemotaxis^4^, and stability of proteins^5,6^. We recently found that lysine 2-hydroxyisobutyrylation (Khib) was involved in the regulation of bacterial metabolism, transcription and antibiotic resistance^7–10^. However, understanding of the function and regulatory mechanism of PTMs in prokaryotes remains limited.

Lysine lactylation (Kla) was recently reported as a newly identified type of PTM on human histones for regulating macrophage polarization and states^11–14^. Further studies showed that Kla on histones can influence cellular metabolic reprogramming during the differentiation of pluripotent stem cells and in non-small cell lung cancer^15,16^, can drive oncogenesis and is associated with neural excitation^17,18^ via the regulation of gene transcription. In addition, Kla on the nonhistone protein HMGB1 has been shown to promote exosomal release in polymicrobial sepsis^19^. To data, Kla has been reported in eukaryotes, including humans, mice, rice, and *Botrytis cinerea*^11,18,20,21^. However, it remains unclear whether Kla exists in prokaryotes and what roles these undiscovered Kla modifications might play.

The dynamic changes of PTMs with time and space are believed to be important biological events, modulating protein function in diverse cellular processes. Reversible PTMs are usually regulated by two types of enzymes that specifically add or remove PTMs. For example, lysine acetyltransferases (KATs) and lysine deacetylases (KDACs) have been extensively reported to catalyze diverse acylation or deacylation^1,22–24^. For Kla, P300 functions as the potential lactylase, and class I histone deacetylases (HDAC1‒3) serve as delactylases^11,25^. However, the regulatory enzymes for Kla in prokaryotes are still unknown.

Here, we profiled the Kla proteome in *Escherichia coli* (*E. coli*) MG1655, identified the writer and eraser for Kla, and illustrated the Kla-mediated effect on bacterial glycolysis and growth. We identified YiaC as a lactylase and CobB as a delactylase by screening strains with overexpressed GCN5-related N-acetyltransferase (GNAT) family proteins and CobB. Furthermore, we revealed that YiaC and CobB can catalyze the addition and removal of lysine lactylation, respectively, both in vitro and intracellularly. Next, by carrying out stable isotope labeling by amino acids in cell culture (SILAC)–based quantitative proteomics approach, we identified 79 endogenous Kla sites potentially regulated by YiaC and 446 Kla candidates targeted by CobB. We identified a total of 1047 Kla sites on 478 proteins in *E*. *coli* MG1655 and showed that lactylated proteins were mostly related to metabolism. We further demonstrated that CobB can erase PykF K382la to enhance glycolysis and promote the growth of *E. coli*. In summary, this study identifies the regulatory enzyme system for Kla in bacteria, profiles the endogenous substrate proteins of Kla, and illustrates the molecular mechanism of Kla-mediated regulation of glycolysis.

## Results

### Identification of YiaC and CobB as the regulatory enzymes for Kla

Kla was originally identified as a histone marker in eukaryotic cells^11–14^. However, it remained unclear whether the novel PTM existed in prokaryotes. To achieve this goal, we performed a western blot assay with pan-Kla antibody for whole-cell lysates of *E. coli*. As shown in Supplementary Fig. 1a, a number of lysine lactylated proteins were detected, confirming the existence of Kla in prokaryotes.

Reversible PTMs that are regulated by writers and erasers modulate protein functions in diverse cellular processes. Therefore, it is imperative to identify the regulatory enzymes of Kla to understand its function and regulatory mechanism in prokaryotes. Previous studies have shown that acetyltransferases (KATs) and deacetylases can regulate diverse lysine acylations, such as acetylation, succinylation and crotonylation^24,26–30^. Therefore, we rationally hypothesized that some KATs and deacetylases may catalyze the addition and removal of Kla.

**Fig. 1:**
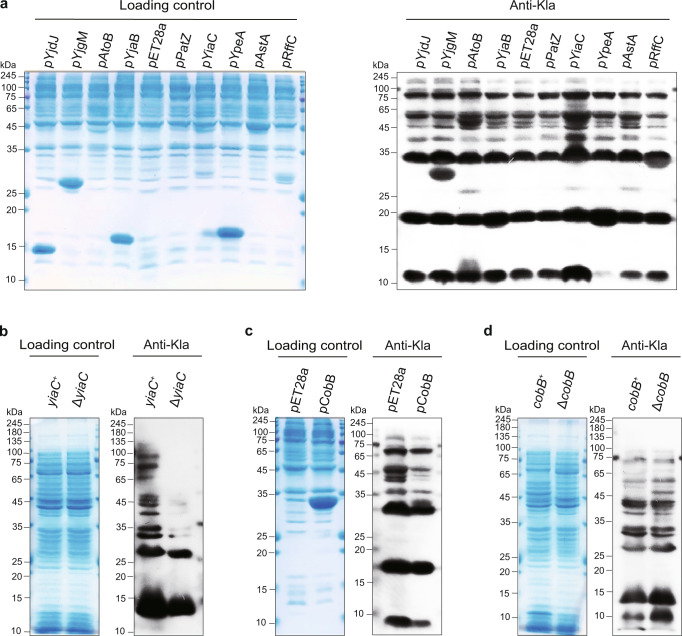
Identification of regulatory system for Kla in *E. coli*. **a** Overexpression of YiaC increased Kla levels in *E. coli*. *E. coli* BL21 (λDE3) was transferred with the empty vector (pET28a) as control and with vector GNAT *genes*-pET28a as GNAT-overexpressing strains (pGNAT), the Kla level of whole-cell lysates was analyzed by immunoblotting. **b** Deficiency of YiaC decreased Kla in *E. coli*. Kla level of *E. coli* MG1655 Δ*yiaC* was analyzed by immunoblotting, with *yiaC*^*+*^ as control. **c** Overexpression of CobB decreased Kla levels in *E. coli*. Immunoblotting analyzed Kla levels of *E. coli* BL21 (λDE3) transferred pET28a as control and *cobB*-pET28a as pCobB strain. **d** Deficiency of CobB increased Kla in *E. coli*. Kla level of *E. coli* MG1655 Δ*cobB* was analyzed by immunoblotting, with *cobB*^*+*^ as control. All immunoblots had three biological repetitions, with similar results. And all strains were cultured in LB medium at 37 °C.

GNATs are the only protein family that act as lysine acetylases in *E. coli*^31,32^. Our recent work showed that TmcA serves as a lysine 2-hydroxyisobutyryltransferase by screening GNAT-overexpressing *E. coli* BL21 (λDE3) strains^9^. Here, we employed 18 GNAT-overexpressing *E. coli* BL21 (λDE3) strains (pGNAT) to screen lactylase candidates by immunoblotting whole-cell lysates with a pan-Kla antibody. Compared with *E. coli* harboring the empty pET28a vector, we found that only overexpressing YiaC caused an extensive and obvious increase in Kla levels (Fig. 1a, Supplementary Fig. 1b, c), identifying YiaC as a lysine lactylase candidate. To confirm its catalytic activity toward Kla, we further detected Kla levels in *E. coli* MG1655 *yiaC*^*+*^ and Δ*yiaC*. The immunoblot results showed that the level of Kla in Δ*yiaC* strain was significantly decreased (Fig. 1b). Together, our results suggest that YiaC is a candidate lactylase that catalyzes the generation of Kla.

To explore the delactylase for *E. coli*, we analyzed the Kla levels of the CobB-overexpressing *E. coli* BL21 (λDE3) strain (pCobB), where CobB is the main known deacylase in *E. coli* for the removal of acetylation, succinylation and 2-hydroxyisobutyrylation^8,33–35^. Compared with *E. coli* harboring the empty pET28a vector, the pCobB strain caused a significant decrease in Kla levels (Fig. 1c). Subsequently, we found that the Kla levels were slightly increased in *E. coli* MG1655 Δ*cobB* compared with *cobB*^*+*^ (Fig. 1d). These results indicated that CobB functions as an endogenous delactylase.

### Regulation of Kla by YiaC and CobB in vitro

It is known that acetyl-CoA or acetyl-phosphate can act as the acetyl group donor for acetylation in prokaryotes^36^. However, there is no evidence of the presence of lactyl-phosphate in *E. coli*. For lactyl-CoA, a previous study suggested that fresh *E. coli* protein extract can catalyze lactate to lactyl-CoA^37,38^. To confirm it, we detected lactyl-CoA in whole-cell lysates as a control. As expected, we detected a low level of lactyl-CoA (Supplementary Fig. 2e), showing that lactyl-CoA is present in *E. coli*. Next, we incubated lactate with fresh whole-cell lysates and found that lactate could be effectively converted to lactyl-CoA (Supplementary Fig. 2e).

**Fig. 2:**
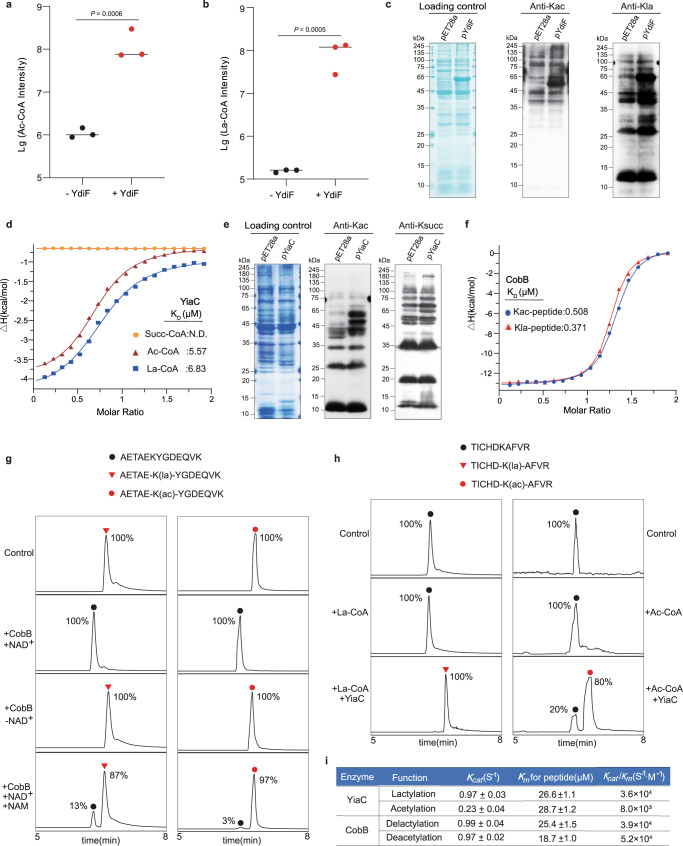
YiaC and CobB regulate Kla in vitro. **a** is performed produce acetyl-CoA (Ac-CoA) by YdiF. − YdiF: incubation acetate with acetoacetyl-CoA (Acac-CoA), + YdiF: incubation acetate with acetoacetyl-CoA (Acac-CoA) with YdiF. *n* = 3 biological repetitions. **b** is performed produce lactyl-CoA (la-CoA) by YdiF. − YdiF: incubation lactate with acetoacetyl-CoA (Acac-CoA), + YdiF: incubation lactate with acetoacetyl-CoA (Acac-CoA) with YdiF. *n* = 3 biological repetitions. **c** YdiF-overexpressing increased the formation of acetyl-CoA and lactyl-CoA. Immunoblotting analyzed Kac and Kla of *E. coli* BL21 (λDE3) transferred pET28a as control and *ydiF*-pET28a as pYdiF strain. **d** ITC analysis of the affinity of recombinant YiaC with lactyl-CoA, acetyl-CoA and succinyl-CoA, three biological repetitions, with similar results. **e** YiaC catalyzed acetylation but not succinylation. Immunoblotting analyzed Kac and Ksucc of *E. coli* BL21 (λDE3) transferred pET28a as control and *yiaC*-pET28a as pYiaC strain. **f** ITC analysis of the affinity of recombinant CobB with Kla peptide (AETAEK(la)YGDEQVK) and Kac peptide (AETAEK(ac)YGDEQVK), three biological repetitions, with similar results. **g** CobB catalyzed delactylation of peptide in vitro. Synthetic peptides (AETAEK(la)YGDEQVK, AETAEK(ac)YGDEQVK) were used as the substrates for CobB reaction with LC-MS detection of lysine delactylation and deacetylation activities of CobB, three biological repetitions, with similar results. **h** YiaC catalyzed lactylation of peptide in vitro. Synthetic peptide (TICHDKAFVR) was used as the substrate for YiaC reaction with LC-MS detection of lysine lactylation and acetylation activities of YiaC, three biological repetitions, with similar results. **i** Kinetic parameters (*kcat*, *Km*, and *kcat/Km*) of YiaC for lactylaiton and CobB for delactylation, *n* = 3 biological repetitions. Data are means ± SEM from three independent assays, two-tailed Student’s *t*-test. *P*-values are indicated in the figure. All immunoblots had three biological repetitions, with similar results. All strains were cultured in LB medium at 37 °C. Source data are provided as a Source Data file.

To further understand the formation of lactyl-CoA, we explored the potential synthetase or transferase of lactyl-CoA. First, we selected acetyl-CoA synthetase (Acs) and propionate CoA synthetase (PrpE) as candidates for lactyl-CoA synthetase in *E. coli*. To examine whether Acs or PrpE can catalyze lactate to lactyl-CoA, we incubated purified Acs with acetate or lactate and PrpE with propionate or lactate, respectively. However, we observed that neither Acs nor PrpE could catalyze the formation of lactyl-CoA from lactate (Supplementary Fig. 2c, d, f, g). It has been recently reported that five CoA transferases from different species, including *Megasphaera elsdenii*, *Clostridium propionicum, Megasphaera* sp. DISK 18, *Clostridium lactatifermentans* An75 and *Firmicutes bacterium* CAG:466 can convert lactate to lactyl-CoA^39,40^. After a BLAST search, we found that acetate CoA-transferase (Ydif) in *E. coli* has high sequence conservation with the five transferases, especially in motifs EXGXXG and GXGGF (Supplementary Fig. 2a, b). Thus, we incubated purified YdiF with acetate or lactate. Interestingly, we found that YdiF could catalyze the conversion of acetyl-CoA or lactyl-CoA from acetate or lactate in vitro (Fig. 2a, b), suggesting that Ydif has lactyl-CoA-transferase activity. To confirm its intracellular catalytic activity, we further analyzed Kac and Kla levels in the YdiF-overexpressing *E. coli* BL21 (λDE3) strain (pYdiF). Compared with *E. coli* harboring the empty pET28a vector, we found that the pYdiF strain caused a significant increase in Kac and Kla levels (Fig. 2c). Together, our results demonstrate that lactyl-CoA does exist in *E. coli* and that YdiF can serve as a lactyl-CoA-transferase for the conversion of lactyl-CoA from lactate.

Previous studies showed that lysine acylases usually bind with relevant acyl-CoA to start the catalytic function^22,41^, while deacylases need to recognize the corresponding modified lysine to catalyze removal of the PTM^8^. Thus, we performed an isothermal titration calorimetry (ITC) assay to detect the binding of YiaC with lactyl-CoA and CobB with lactylated peptide. Because YiaC is a known lysine acetylase and CobB is a deacetylase^32,33^, we separately used acetyl-CoA and acetylated peptide as controls. As anticipated, there was strong binding between YiaC and lactyl-CoA (*K*_*D*_ = 6.83 μM), which was similar to acetyl-CoA (*K*_*D*_ = 5.57 μM); instead, there was no binding between YiaC and succinyl-CoA (Fig. 2d, Supplementary Data 1). Next, we found that YiaC overexpression could upregulate Kac as reported^32^ but did not cause a change in succinylation compared with *E. coli* transformed with the pET28a vector (Fig. 2e), which was consistent with the ITC results. These results provide further supporting evidence that YiaC can catalyze the addition of Kla. We also detected the binding of CobB with lactylated peptide (AETAEK(la)YGDEQVK, from GpmA) and acetylated peptide (AETAEK(ac)YGDEQVK, from GpmA). The ITC results showed that CobB could bind with lactylated peptide (*K*_*D*_ = 0.371 μM), similar to acetylated peptide (*K*_*D*_ = 0.508 μM) (Fig. 2f, Supplementary Data 1).

To characterize the enzymatic activities of YiaC and CobB, we further performed enzyme reaction assays in vitro with synthetic peptides. Liquid chromatography–MS (LC-MS) was employed to monitor Kla and Kac of the reacted peptides, and the MS/MS spectrum was used to verify its veracity. By incubating CobB with lactylated or acetylated peptide (AETAEK(la or ac)YGDEQVK, from GpmA), we found that CobB efficiently catalyzed the hydrolysis of the lactyl peptide in the presence of NAD^+^ (nicotinamide adenine dinucleotide). In contrast, the delactylated peptide was not detected in the reaction without NAD^+^, suggesting an NAD-dependent delactylation mechanism, similar to deacetylation (Fig. 2g). Subsequently, we found that the delactylation reaction could be inhibited by nicotinamide (NAM) (a class III HDAC inhibitor) and that NAM was more effective for deacetylation than delactylation (Fig. 2g). These results indicated that NAD-dependent CobB can catalyze lysine delactylation in vitro, which can be inhibited efficiently by NAM. Moreover, by incubating YiaC with a peptide (TICHDKAFVR) presenting lactyl-CoA or acetyl-CoA, we found that YiaC can catalyze lactylation in vitro. Compared to acetylation, YiaC is likely more efficient for lactylation (Fig. 2h).

To determine the enzymatic kinetics of YiaC and CobB, we next used a matrix-assisted laser desorption/ionization–time-of-flight (MALDI-TOF) MS assay to calculate the kinetic parameters (*kcat, Km*, and *kcat/Km*) of YiaC for lactylation and CobB for delactylation. The kinetic measurements (Fig. 2i, Supplementary Fig. 3, Supplementary Data 2) confirmed the lactylase activity of YiaC (*k*_cat_ = 0.97 ± 0.03 s^−1^, *K*_m_ = 26.6 ± 1.1 μM, *k*_cat_ /*K*_m_ = 3.6 × 10^4^ s^−1.^M^−1^) is more effective than acetylase (*k*_cat_ = 0.23 ± 0.04 s^−1^, *K*_m_ = 28.7 ± 1.2 μM, *k*_cat_ /*K*_m_ = 8.0 × 10^3^ s^−1.^M^−1^). The delactylase activity of CobB (*k*_cat_ = 0.99 ± 0.04 s^−1^, *K*_m_ = 25.4 ± 1.5 μM, *k*_cat_ /*K*_m_ = 3.9 × 10^4^ s^−1.^M^−1^) is similar to the deacetylase activity (*k*_cat_ = 0.97 ± 0.02 s^−1^, *K*_m_ = 18.7 ± 1.0 μM, *k*_cat_ /*K*_m_ = 5.2 × 10^4^ s^−1.^M^−1^). These results show the enzymatic kinetics characterization of YiaC for lactylation and CobB for delactylation.

### Identification of the endogenous substrates of Kla targeted by YiaC and CobB in *E. coli*

**Fig. 3:**
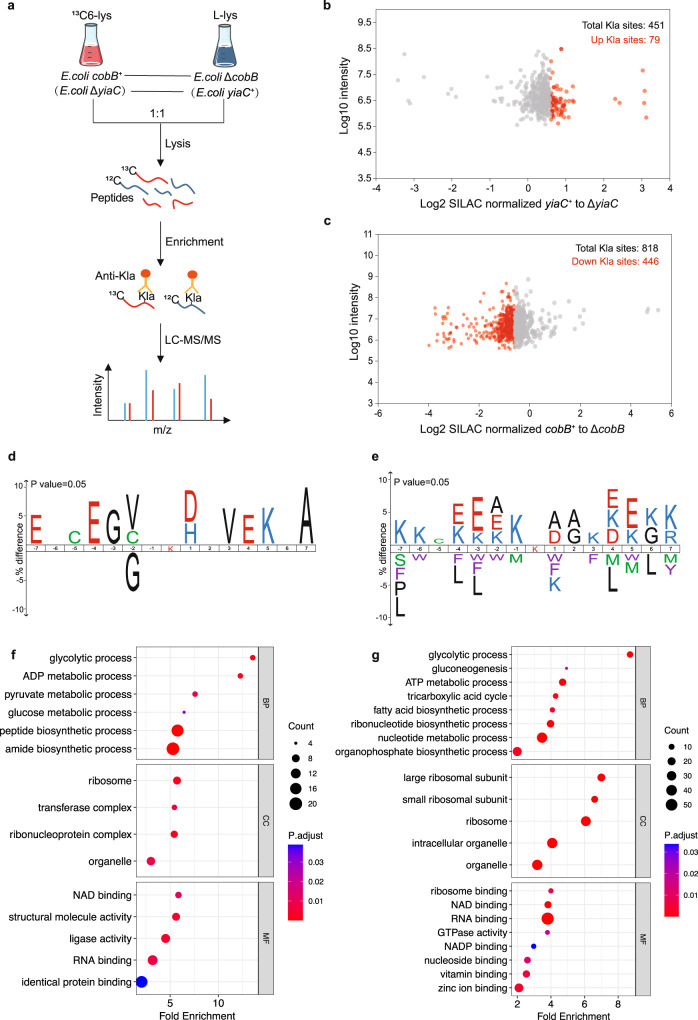
Profiling endogenous substrate proteins for Kla by Yiac and CobB, respectively, in *E. coli*. **a** Schematic representation of experimental workflow for SILAC quantification. **b** Scatterplot showing the ratio of Kla peptides in *E. coli* MG1655 *yiaC*^*+*^ versus Δ*yiaC* (normalized by protein abundance). **c** Scatterplot showing the ratio of Kla peptides in *E. coli* MG1655 *cobB*^*+*^ versus Δ*cobB* (normalized by protein abundance). **d** Sequence motif logo shows a representative sequence for YiaC-regulated Kla sites. **e** Sequence motif logo shows a representative sequence for CobB-regulated Kla sites. **f** GO analysis for Kla protein candidates targeted by YiaC. **g** GO analysis for Kla protein candidates targeted by CobB. The *p*-value cutoff = 0.05 and q-value cutoff = 0.2 were selected as the cutoff criteria. Benjamini and Hochberg correction was used to adjust *P*-values.

To understand the endogenous Kla substrates regulated by YiaC and CobB, we performed quantitative proteomics by combining SILAC and affinity enrichment to analyze the influence of YiaC or CobB gene deletion on the lactylome in *E. coli* (Fig. 3a). Comparing the *E. coli* MG1655 *yiaC*^*+*^ and Δ*yiaC* strains, we quantified 451 Kla sites in 247 proteins (Supplementary Data 3). Normalizing the detected abundances to the expression of their corresponding proteins (Supplementary Fig. 4a), we quantified 79 significantly downregulated Kla sites (more than 1.5x reduction) in *E. coli* Δ*yiaC* (Fig. 3b). These downregulated Kla may be potentially functional YiaC-targeted substrates. Moreover, comparing the *E. coli* MG1655 *cobB*^*+*^ and Δ*cobB* strains, we quantified 818 Kla sites in 375 proteins (Supplementary Data 4). Normalized by the abundance of corresponding proteins (Supplementary Fig. 4b), the result showed that more than the half of Kla sites were significantly upregulated (more than a 1.5× increase) in the Δ*cobB* strain compared with the *cobB*^*+*^ strain (Fig. 3c). A total of 446 upregulated Kla sites were candidate CobB-targeted substrates. By comparing the candidates targeted by YiaC and CobB, we found that 63% proteins lactylated by YiaC were also substrates for CobB (Supplementary Fig. 4c), while only 29 Kla sites on 25 proteins were markedly coregulated by these two enzymes (Supplementary Fig. 4d), which indicates that the Kla regulatory system composed of YiaC and CobB may not be the only one, and it is likely that there are other intracellular regulatory enzymes. Furthermore, we analyzed the sequence characteristics of Kla peptides potentially regulated by YiaC and CobB. As shown in Fig. 3d, acidic glutamate and aspartate and hydrophobic valine clearly surrounded Kla located in YiaC-targeted Kla peptides. In addition, acidic glutamate, hydrophobic alanine and basic lysine surrounded Kla located in CobB-targeted Kla peptides (Fig. 3e). The difference in sequence characteristics indicated that YiaC and CobB tend to catalyze Kla in different characteristic amino acid sequences. This also explains to some extent why only a small number of common regulated sites were identified. To understand the biological significance of YiaC and CobB regulation, we further performed Gene Ontology (GO) analysis of Kla protein candidates targeted by YiaC or CobB. We found that Kla candidates for YiaC or CobB are both mainly related to metabolism and biosynthesis, especially glycolysis, and mainly occur in the ribosome and cytosol with diversity binding activities (Fig. 3f, g), suggesting that YiaC- and CobB-regulated Kla may influence metabolism and biosynthesis.

### Systematic analysis of lysine lactylome in *E. coli*

**Fig. 4:**
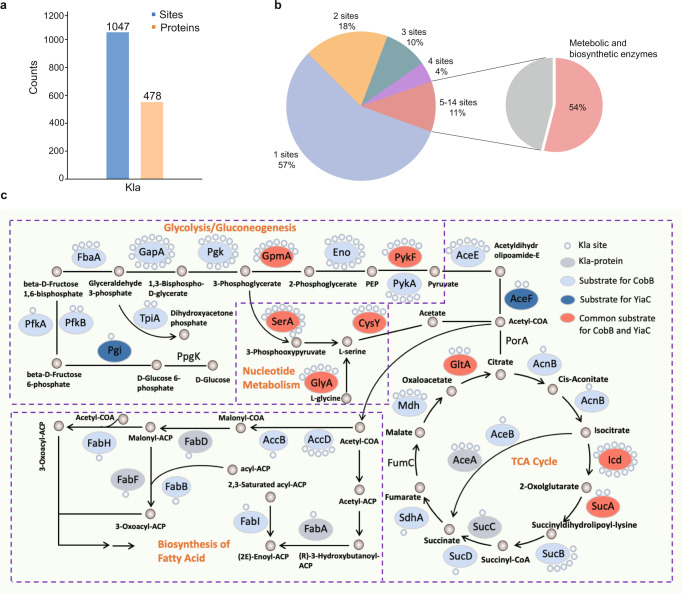
Characterization of the Kla proteome in *E. coli*. **a** Statistics analysis of the Kla proteins and sites of *E. coli*. **b** The frequency of Kla occurs on proteins. **c** The lactylated enzymes in central carbon metabolism and fatty acid biosynthesis network in *E. coli*.

The biological characteristics of Kla in prokaryotes remained unclear, so we combined the two groups of quantified lactylome data listed in Supplementary Data 3, 4 and selected all identified Kla data in wild-type *E. coli* MG1655 as the lactylome in *E. coli*. We identified a total of 1047 Kla sites in 478 proteins (Fig. 4a, Supplementary Data 5). Analyzing the frequency of Kla in proteins, we found that most identified proteins (~57%) have 1–2 Kla sites, and 11% of proteins have more than 5 Kla sites. Interestingly, many proteins rich in Kla sites are metabolic and biosynthetic enzymes (Fig. 4b), indicating that Kla may have functional effects on bacterial metabolism and biosynthesis. Analyzing the sequence characteristics of Kla peptides, we found that acidic glutamate and basic lysine were surrounded by Kla with high confidence (Supplementary Fig. 5a). Furthermore, GO analysis of this lactylome showed that lactylated proteins are mainly related to metabolism, translation, ribosome assembly and biosynthesis and mainly occur in ribosomes with diverse binding activities (Supplementary Fig. 5b). To analyze the probability of Kla relative to its substrate, we used the intensity of Kla identified in wild-type *E. coli* MG1655 divided by the intensity of the corresponding protein. We defined a ratio >0.001 as Kla sites with a high frequency on the proteins (Supplementary Data 6); thus, a total of 323 Kla sites on 240 proteins were defined as occurring with high frequency (Supplementary Fig. 5c, d). GO analysis showed that these proteins with high frequency Kla are also mostly related to various metabolic pathways (Supplementary Fig. 5e). Notably, further analysis of lactylated proteins in pathways revealed that almost all of the enzymes in glycolysis, tricarboxylic acid cycle (TCA) and fatty acid biosynthesis are lactylated; moreover, most of these enzymes were also identified as candidates for CobB or YiaC regulation (Fig. 4c). It was also shown that CobB has a wider regulatory range than YiaC, and they can jointly regulate the same protein and have their own specific regulatory proteins.

### YiaC and CobB mediate Kla to regulate the enzymatic activities of substrate proteins in vitro

**Fig. 5:**
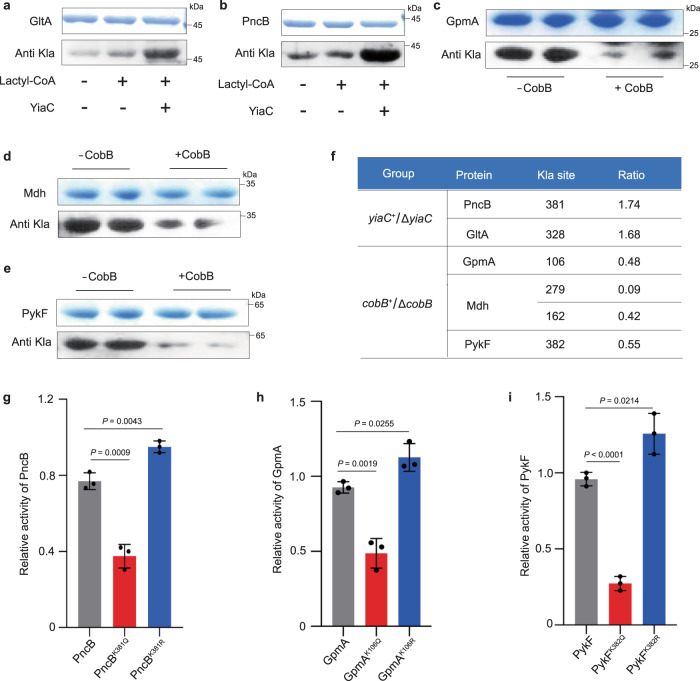
YiaC and CobB regulation Kla of multiple enzymes and influence enzymatic activities in vitro. **a**, **b** YiaC can specifically catalyze Kla of GltA and PncB in vitro. Recombinant GltA or PncB was incubated with YiaC in the presence of lactyl-CoA (1 mM) for 10 h at 25 °C, and immunoblotting was performed. **c**–**e** CobB can specifically erase Kla of GpmA, Mdh, and PykF in vitro. Recombinant GpmA, Mdh, or PykF was incubated with CobB for 10 h at 25 °C, and immunoblotting was performed. **f** The list of identified Kla sites of enzymes. **g**–**i** Detection of the activities of enzymes and their mutants (K to Q and K to R) (data are means ± SEM from three independent assays, two-tailed Student’s *t*-test). All immunoblots had three biological repetitions, with similar results. Source data are provided as a Source Data file.

To explore the function of Kla regulated by YiaC and CobB, we performed in vitro experiments. According to the Kla networks (Fig. 3b, c), we further performed a group of in vitro reactions in which YiaC and CobB were incubated with their respective candidate proteins. Then, Kla levels of candidate proteins were detected by immunoblotting. We found that YiaC can effectively lactylate citrate synthase (GltA) and nicotinate phosphoribosyltransferase (PncB) (Fig. 5a, b), while CobB can clearly delactylate pyruvate kinase I (PykF), 2,3-bisphosphoglycerate-dependent phosphoglycerate mutase (GpmA) and malate dehydrogenase (Mdh) in vitro (Fig. 5c–e). Thus, it can be seen that YiaC and CobB can regulate the Kla levels of diverse metabolic and biosynthetic enzymes; in particular, CobB can widely delactylate glycolysis enzymes in vitro, which is consistent with the results of GO analysis shown in Fig. 3g.

To determine whether Kla can influence enzymatic activity. First, we confirmed the Kla sites of these proteins regulated by YiaC and CobB from Supplementary Data 3 and 4 (Fig. 5f), and then we replaced lactylated K by Q (glutamine) because the electrical property of Kla is neutral, similar to that of glutamine and replaced unmodified K by R (arginine) because the positive charge of K is similar to that of R. Combining the enzymatic activity assays in vitro, we found that the enzymatic activities of simulating the Kla variants PncB^K381Q^, GpmA^K106Q^, and PykF^K382Q^ were significantly decreased compared to those of the wild-type proteins. The enzymatic activities of the unmodified mutants PncB^K381R^, GpmA^K106R^, and PykF^K382R^ were increased (Fig. 5g–i). These results suggest that YiaC and CobB can mediate Kla to influence the activities of metabolic enzymes.

### CobB regulates PykF activity and influences cell growth by erasing K382la

**Fig. 6:**
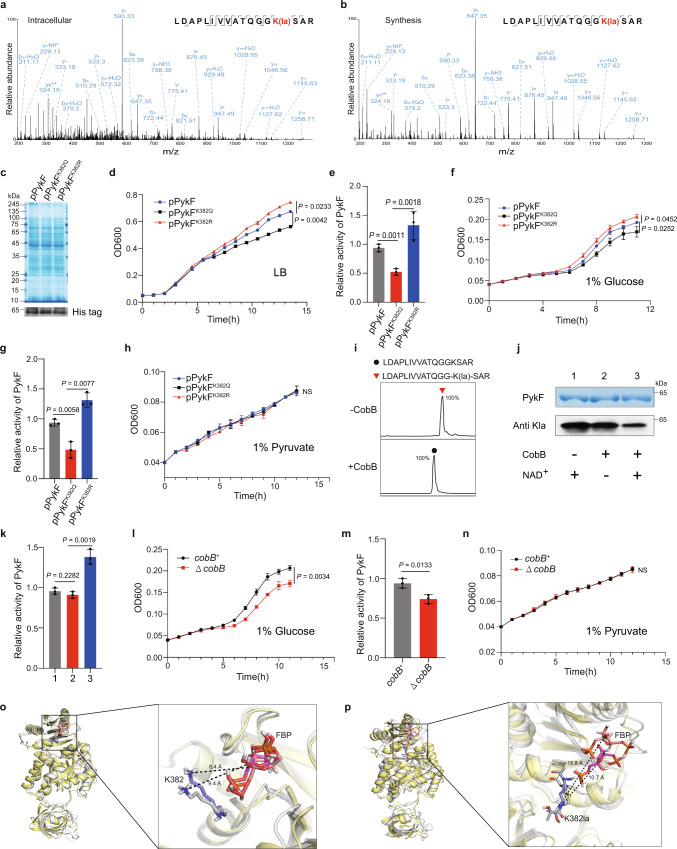
CobB influence PykF activity, glycolysis, and cell growth by erasing K382la. **a** The MS/MS spectrum of peptide (LDAPLIVVATQGGK(la)SAR, PYKF) from proteome of Δ*cobB*. **b** The MS/MS spectrum of synthetic peptide (LDAPLIVVATQGGK(la)SAR). **c** Selection the same expression of recombinant PykF for overexpression strains by immunoblotting with his tag antibody. **d** Measurement growth curve of PykF-overexpressing *E. coli* strains (pPykF, pPykF^K382Q^, and pPykF^K382R^) cultured in LB medium at 37 °C with 96-well plates. **e** Measurement of the intracellular activity of PykF of strains related to **d**. **f** Measurement growth curve of pPykF, pPykF^K382Q^, and pPykF^K382R^ strains cultured in M9 medium with 1% glucouse at 37 °C with 96-well plates. **g** Measurement of the intracellular activity of PykF of strains related to **f**. **h** Measurement growth curve of pPykF, pPykF^K382Q^, and pPykF^K382R^ strains cultured in M9 medium with 1% pyruvate at 37 °C with 96-well plates. **i** CobB catalyzed delactylation of PykF K382la in vitro. Synthetic PykF K382la peptide (LDAPLIVVATQGGK(la)SAR) was used as the substrate for CobB reaction with LC-MS detection of lysine delactylation activity of CobB. **j**, **k** CobB regulation PykF activity by erasing K382la in vitro. PYKF was incubated with CobB to decrease K382la (**j**), after delactylation, detection of the activity of PykF (**k**). **l** Measurement growth curve of *E. coli* MG1655 *cobB*^*+*^ and Δ*cobB* strains cultured in M9 medium with 1% glucose at 37 °C with 96-well plates. **m** Measurement of the intracellular activity of PykF of strains related to **l**. **n** Measurement growth curve of *E. coli* MG1655 *cobB*^*+*^ and Δ*cobB* strains cultured in M9 medium with 1% pyruvate at 37 °C with 96-well plates. **o** 3D analysis of PykF-FBP system MD simulation result. **p** 3D analysis of PykF K382la-FBP system MD simulation result. For measuring PykF activity and growth curve, *n* = 3 biological replicates, data are means ± SEM, two-tailed Student’s *t*-test, *P*-values are indicated in the figure, NS means not significant. OD600, optical density at 600 nm. Immunoblots had three biological repetitions, with similar results. Source data are provided as a Source Data file.

Notably metabolic and synthetic pathways affect bacterial growth^42–44^. Therefore, we rationally predicted that the changes in PncB, GpmA and PykF activities caused by Kla may influence bacterial growth. To confirm this hypothesis, we first confirmed the reliability of the identified Kla peptides by synthesizing three kinds of Kla peptides from the YiaC-related proteome and three kinds of Kla peptides from the CobB-related proteome containing the peptides PncB K381la (TICHDK(la)AFVR), GpmA K106la (AETAEK(la)YGDEQVK), and PykK382la (LDAPLIVVATQGGK(la)SAR). As shown in Fig. 6a, b and Supplementary Fig. 6, the entire MS/MS spectra of synthetic Kla peptides overlap almost completely with intracellularly modified peptides; therefore, the reliability of the Kla peptides was confirmed. Next, we separately constructed PykF, PykF^K382Q^, and PykF^K382R^ overexpressing *E. coli* MG1655 (pPykF, pPykF^K382Q^, and pPykF^K382R^ strains), and we constructed the pPncB, pPncB^K381Q^, pPncB^K381R^, pGpmA, pGpmA^K106Q^, and pGpmA^K106R^ strains. Then, the growth curves were detected after culturing in lysogeny broth (LB) medium. We found that pPncB, pGpmA and their mutant strains showed no difference in growth (Supplementary Fig. 7a, b). However, in the PykF group, the pPykF^K382R^ strain had the fastest growth, while the pPykF^K382Q^ strain grew the slowest among the three strains with the same overexpression levels (Fig. 6c, d). Furthermore, we detected intracellular PykF activity. Consistent with the growth curves, the pPykF^K382R^ strain showed the highest activity and pPykF^K382Q^ had the lowest activity among the three strains (Fig. 6e). As reported, PykF is a critical rate-limiting enzyme in glycolysis that controls metabolic flux and influences bacterial cell division^45^. These results demonstrate that PykF K382la slows bacterial growth by reducing PykF activity. To further confirm this observation, considering that phosphoenolpyruvate produced by glucose glycolysis can produce pyruvate under the catalysis of PykF^46,47^, we selected M9 minimal medium with 1% glucose to make PykF perform its normal metabolic function and selected M9 minimal medium with 1% pyruvate to block the function of PykF. Then, we measured the growth curves and intracellular PykF activity of these three strains in M9 minimal medium with 1% glucose, as in LB. The pPykF^K382R^ strain grew fastest and showed the highest PykF activity, and the pPykF^K382Q^ strain grew slowest and showed the lowest PykF activity (Fig. 6f, g). Conversely, to block the influence of PykF, we measured the growth curves and intracellular PykF activity of these three strains cultured in M9 minimal medium with 1% pyruvate. We found that there was no difference in the growth of the three strains (Fig. 6h). These results illustrate that PykF K382la affects bacterial growth by reducing PykF activity.

The lactylome showed that CobB delactylated PykF K382la intracellularly (Fig. 5f). To confirm this result, we incubated PykF K382la peptide (LDAPLIVVATQGGK(la)SAR) with CobB and detected the products by LC‒MS/MS, and the results showed that CobB can effectively catalyze the delactylation of PykF K382la in vitro (Fig. 6i). Thus, we incubated PykF with CobB to erase PykF K382la (Fig. 6j) and then detected the activity. The result showed that CobB erasing PykF K382la enhanced PykF activity in vitro (Fig. 6k). For intracellular assays, we measured the growth curves and intracellular PykF activity of *cobB* + and Δ*cobB* strains cultured in M9 minimal medium with 1% glucose or 1% pyruvate. Furthermore, we found that the *cobB* + strain grew faster than the Δ*cobB* strain in M9 minimal medium with 1% glucose (Fig. 6l). Moreover, the intracellular PykF activity of *cobB* + was higher than that of the Δ*cobB* strain (Fig. 6m). As expected, there was no difference in the growth of the *cobB* + and Δ*cobB* strains (Fig. 6n). Together, these results demonstrate that CobB can regulate PykF activity and influence bacterial growth by erasing PykF K382la in vitro and intracellularly.

### PykF K382la affects the allosteric regulation of PykF

Previous studies indicated that fructose 1,6-bisphosphate (FBP) can bind with PykF and allosterically activate PykF, and K382 is a key site-related allosteric regulation of PykF^48–50^. Therefore, we speculated that the decrease in PykF activity by K382la may be due to the influence on the allosteric activation. To confirm this, we further performed 100 ns molecular dynamics (MD) simulations for PykF-FBP and PykF K382la-FBP for three times, respectively. To observe the dynamic stability of the two system complexes, root mean square deviations (RMSDs) were monitored from the starting structure. We found that the average RMSDs of these two system complexes fluctuate slightly (Supplementary Fig. 8a), suggesting that both systems are stable. To better understand the influence on the binding, we measured the average distances between the nitrogen atom of K382 side chain and the central carbon atom of FBP in both PykF-FBP system and PykF K382la-FBP system from 45 ns to 100 ns, respectively (Supplementary Fig. 8b). We found that the average distance of three times between the K382 and FBP is very stable in PykF-FBP system, but that of the K382la and FBP have significant fluctuations in the PykF K382la-FBP system. This result indicates that PykF K382la may affect the binding between FBP and PykF. In order to observe the changes in the microstructure, we selected the initial and stable structure (0 and 55 ns) using the GROMACS on two simulation processes, respectively. For the PykF-FBP system, we can see that the distance of K382 and FBP in the initial structure is 9.4 Å, and that in the stable structure is only 8.4 Å (Fig. 6o). For the PykF K382la-FBP system, the distance of the K382la and FBP in the initial structure is 10.7 Å, while that in the stable structure is 15.8 Å (Fig. 6p). Thus, these results suggested that PykF K382la hindered the binding between FBP and PykF and further weakened the allosteric activation of PykF, finally reducing the activity of PykF.

## Discussion

Lactate, formerly considered metabolic waste, has now been recognized as an important metabolite in mammals. Lactate acts as a major circulating carbohydrate fuel by providing three-carbon compounds^51^ and modulating cellular microenvironments to regulate tumor development and immune response^52,53^. The discovery of lysine lactylation opened the horizon of functional regulation by lactate, and Kla on histones served as a significant marker to regulate transcription in eukaryotes^11,15,17^. All this evidence demonstrated the importance of Kla. For bacteria, lactate was initially identified as a universal secondary metabolite, and bacterial fermentation is the main method of the industrial production of lactate^54^. Lactate serves as an important carbon source for bacterial metabolism^55^, and microbial lactate utilization can modulate stress resistance, cell wall remodeling and virulence factors^56^. In addition, host microbial metabolites are a critical factor influencing many physiological processes and the development of diseases^57^, in which lactate can disturb the microenvironment and cause an immune response^56,58^. Whether Kla occurs in prokaryotes and what biological functions it might perform remain unclear. Thus, it is necessary to explore the characterization, functions and regulatory mechanisms of Kla in bacteria.

**Fig. 7:**
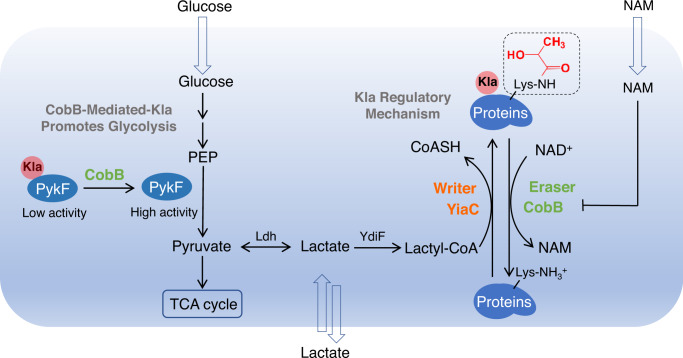
Graphic model as discussed in the text. YiaC and CobB regulated Kla in *E. coli* and influence bacterial growth by regulating PykF activity.

Our study shows that Kla is widely distributed in bacterial proteins. Importantly, we identified a pair of Kla catalytic enzymes in which CobB serves as delactylase, while YiaC functions as lactylase (Fig. 7). By the thermodynamic binding constant and enzyme kinetics, we characterized the catalytic abilities of CobB and YiaC and further proved that the regulatory mechanism of delactylation by CobB is dependent on NAD, and lactylation by YiaC is dependent on La-CoA. For the generation of Kla, we detected the presence of lactyl-CoA as a donor for Kla in *E. coli* and found that fresh whole-cell lysates exhibit catalytic activity for the conversion of lactate to lactyl-CoA (Supplementary Fig. 2e). Furthermore, we identified YdiF as a transferase for the production of lactyl-CoA in *E. coli* (Fig. 2a–c).

To understand the endogenous regulatory networks, we performed quantitative proteomics and identified 79 Kla candidate sites regulated by YiaC and 446 Kla candidate sites targeted by CobB. It is worth noting that YiaC and P300 belong to different protein families, while the latter has lactylase activity in human cells^11^, and CobB is a delactylase, which is also distinct from HDAC1-3, which serve as delactylases in eukaryotes^25^. This finding thereby indicates that there may be other potential lactylases and delactylases in eukaryotes. In addition, YiaC was previously reported as a lysine and N-terminal acetylase^59,60^, and CobB was reported as a lysine acetylase, succinylae and 2-hydroxyisobutyryltransferase^8,33,34^. Therefore, we expanded the understanding of acylation regulatory enzymes in regulatory and functional diversity. Lysine acylation is dose-dependent on the metabolism of acyl-CoA and corresponding organic acids^61,62^, and changes in metabolism can mobilize regulatory enzymes to regulate acylation levels. Lactate accumulates in *E. coli* under glucose fermentation^63^, and the increased lactate level may mobilize YiaC and CobB to maintain the balance of Kla.

Here, we report a global analysis of the lysine lactylomes in bacteria. A total of 1047 Kla sites were identified in 478 proteins in *E. coli*. This modification is widespread but is mainly enriched in metabolic enzymes. As shown in Fig. 4c, almost all of the enzymes in glycolysis and TCA are lactylated; notably, these enzymes are also identified as candidates targeted by CobB, suggesting that CobB may erase Kla to regulate the activities of metabolic enzymes. Indeed, our results show that CobB and YiaC can mediate Kla to exert effects on their substrates. For example, CobB downregulates K106la of GpmA and K382la of PykF to enhance the activities of the corresponding enzymes, and YiaC upregulates K381la of PncB to the decrease activity of PncB. Importantly, we demonstrate that CobB erases K382la of PykF to promote glycolysis and bacterial growth. Our study reveals that Kla has an extensive regulatory effect on metabolism in bacteria, distinct from the regulatory transcription of Kla in eukaryotes^11,15^. As the end product of anaerobic glycolysis in bacteria (Fig. 7)^55^, lactate can be catalyzed to produce lactyl-CoA, which is a necessary substrate for the generation of Kla^11^. Therefore, metabolites can effectively influence the Kla level of intracellular metabolic enzymes. Conversely, our study reveals that CobB-mediated Kla regulates the activities of metabolic enzymes and further influences glycolysis, suggesting a metabolism-PTM-metabolism signaling cascade that regulates the balance of metabolism and PTM.

In summary, we originally identified the prokaryotic Kla regulatory system in which YiaC is a lysine actylase and CobB serves as a delactylase. We subsequently profiled the endogenous candidates of YiaC and CobB, providing a framework to investigate the diverse functions of Kla regulated by YiaC and CobB in prokaryotes. Additionally, we described the characteristics of the lactylome in *E. coli* containing 1047 Kla sites in 478 proteins. Importantly, we demonstrated that CobB can increase the activity of PykF by erasing K382la, enhancing glycolysis and bacterial growth. Our work presents the regulatory system and molecular network of Kla and offers new insight into the Kla-mediated mechanism for the regulation of metabolism in bacteria.

## Methods

### Strains and reagents

*E. coli* MG1655 cells and *E. coli* MG1655 Δ*cobB* cells were obtained from our previous study^8^, *E. coli* BL21 (λDE3) was from TIANGEN, *E. coli* MG1655 Δ*yiaC*, and GNAT family genes overexpressing *E. coli* BL21 (λDE3) strains were obtained from our previous study^9^. All the anti-acyllysine antibodies were purchased from PTM Biolabs Inc. All the synthetic peptides were generated by SciLight Biotechnology, LLC. Antibodies (all diluted 1:1000) and other reagents are listed in Supplementary Data 7.

### Construction of gene overexpressing *E. coli* strains

This method was performed as described previously^9^. In detail, plasmids were constructed to express proteins in *E. coli*. We used pEASY®-Basic Seamless Cloning and Assembly Kit (TRANS) to construct the plasmids of pET28a-*cisY*, pET28a-*pncB*, pET28a-*gpmA*, pET28a-*mdh*, pET28a-*pykF*, and pBR322-*pykF*-His tag. For mutation, we used the Fast Mutagenesis System (TRANS) to construct the vectors containing point mutated genes. All primers used for PCR are listed in Supplementary Data 7. The constructed vectors of pET28a were transformed into *E. coli* BL21 (λDE3) for overexpressing strains. The constructed vectors of pBR322 were transformed into *E. coli* MG1655 and cultured in LB medium containing ampicillin (Amp, 50 μg ml^–1^), at 37 °C in shaken flasks at 220 rpm. overnight.

### Purification of recombinant proteins

The constructed *E. coli* BL21 (λDE3) transformed with *gene*-pET28a vectors were cultured in LB medium containing kanamycin (Kana, 50 μg ml^–1^), at 16 °C in shaken flasks to an optical density of 0.6–0.8 at 600 nm. Next, cells were induced with 0.05 mM IPTG at 16 °C overnight followed by harvesting of whole-cell lysates for immunoblotting or protein purification. The proteins were harvested from cultured cells by lysis buffer (20 mM Tris-HCl, pH 8.0, 10 mM MgCl_2_, 1 mg ml^–1^ lysozyme, 50 U ml^–1^ nuclease). Then the proteins were mixed with HisPur Ni-NTA Resin and washed by wash buffer (20 mM Na_3_PO_4_, 300 mM NaCl, 25 mM imidazole, pH 7.4), the overexpressed proteins were eluted with elution buffer (20 mM Na_3_PO_4_, 300 mM NaCl, 250 mM imidazole, PH 7.4). The elution was collected and concentrated using an Amicon Ultra-0.5 Centrifugal Filter Device in storage buffer (100 mM HEPES, 10 mM MgCl_2_, 7.7 mM KCl, pH 7.0).

### Chemical synthesis of lactyl-CoA

We synthesized lactyl-CoA as described previously^8^. In detail, dicyclohexylcarbodiimide was added to a dimethylformamide mixture solution containing lactic acid and thiophenol, and stirred at 4 °C, the reaction was stopped by adding cold water. The solution was extracted by ether and washed three times with saturated sodium chloride, and dried the ether extract by anhydrous sodium sulfate, then purified the residue by silica gel column chromatography (eluted by a 20:1 mixture of ethyl acetate and hexane) followed by silica thin-layer chromatography (eluted by a 1:4 mixture of ethyl acetate and hexane). The purification was dissolved in 0.1 M sodium bicarbonate, then added to sodium bicarbonate buffer containing sodium salt of CoA at 0 °C. After placing overnight, the reaction was terminated by the HCl to pH 7.0. The final product was evaporated the water at 30 °C, and extracted by ether and ethyl acetate.

### In vitro assay for Acs and PrpE activity

This method was performed as described previously^64^. In detail, the purified Acs or PrpE with a final concentration of 0.3 μM were added in the reaction buffer [50 mM HEPES, 10 mM MgCl_2_, 2.5 mM ATP, 0.5 mM HSCoA, 3 mM phosphoenolpyruvate, 0.1 mM NADH, 1 U pyruvate kinase, 1.5 U lactate dehydrogenase, 5 U myokinase, 10 mM DTT, and 0.2 mM organic acid substrate (acetate, lactate and propionate), PH 7.5] at 25 °C for 30 min. After quenching the reaction by adding equal volume of 10% (v/v) trifluoroacetic acid and cleaning with C18 ZipTips, the products of actyl-CoA were analyzed by LC-MS/MS. Each reaction was performed three biological repetitions.

### Assay for whole-cell lysate reaction

This method was described previously^37^. In detail, *E. coli* MG1655 was cultured in LB medium at 37 °C in shaken flasks overnight and harvested the cells by centrifugation, then added lysate buffer [50 mM NaH_2_PO_4_, 50 mM NaH_2_PO_4_ and 5 mM MgCl_2_, PH 7.0] and broke cells by ultrasound on ice, the supernatant was extracted by high-speed centrifugation as the whole protein lysate. Then measured protein concentration by BCA and adjusted the protein concentration to 2 mg/ml. Adding 5 mM ATP, 2 mM HSCoA, 10 mM DTT and 0.5 mM lactate in whole protein lysate as reaction system, the reaction was performed at 25 °C for 30 min, after quenching the reaction by adding equal volume of 10% (v/v) trifluoroacetic acid and centrifugation, the supernatant was cleaned with C18 ZipTips, and the products of lactyl-CoA were analyzed by LC-MS/MS. Each reaction was performed three biological repetitions.

### In vitro assay for YdiF activity

Assay of the YdiF CoA-transferase activity was performed as described previously^40^, with slight modifications as follows: the enzyme reactions were carried out in phosphate buffer [50 mM NaH_2_PO_4_, 50 mM Na_2_HPO_4_ and 5 mM MgCl_2_, PH 7.0] containing 1 mM acetoacetyl-CoA and 10 mM L-lactate or acetate at 35 °C for 20 min, and initialized by adding the 7.5 μg of purified YdiF. The total volume of the reaction mixture was 50 μL. The mixtures were pre-incubated at the designated reaction temperature for 5 min before the initiation. The reactions were terminated by adding equal volume of 10% (v/v) trifluoroacetic acid and centrifugation, the supernatant was cleaned with C18 ZipTips, and the products of lactyl-CoA were analyzed by LC-MS/MS. Each reaction was performed three biological repetitions.

### HPLC-MS/MS analysis for acyl-CoA

Samples dissolved in water were analyzed by an Orbitrap Exploris 480 (Thermo Scientific) mass spectrometer in positive ESI mode using the settings described previously^65,66^. In detail, samples were separated by a ACQUITY UPLC BEH C18 column (2.1*100 mm,1.7 μm), the HPLC gradient for 15 min was set up as follows: 0.2 mL/min flow at 98% buffer A (water with 5 mM ammonium acetate) and 2% buffer B (95 % acetonitrile in water with 5 mM ammonium acetate) for 0 min, 98 to 70% buffer A for 8 min, 70 to 2% buffer A for 1 min, 2% buffer A for 3 min, 2 to 98% buffer A for 1 min, 98% buffer A for 2 min. An Orbitrap Exploris 480 (Thermo Fisher Scientific) was employed for MS analysis. Spray voltage was set to 3.5 kV, funnel RF level at 40, and ion transfer tube temperature at 320 °C. Data were collected by Xcalibur (v.4.0.27.19). The orbitrap mass analyzer was used as the MS1 detector with 120,000 resolution. The normalized AGC target and maximum injection time were set at 70% /standard for MS1. The MS2 detector was used 15,000 resolution. Targeted mass setting is lactyl-CoA: 840.1436, acetyl-CoA: 810.1330 and propionate CoA: 824.1487. MS2 isolation window was 2 Da and mass tolerance was 10 ppm, and a normalized HCD (higher-energy collision-induced dissociation) collision energy of 25 % was used for precursor fragmentation. The relative intensity of acyl-CoA was analyzed by Xcalibur (v.4.0.27.19).

### ITC measurement

This method was described previously^8^. In detail, using the MicroCal PEAQ-ITC titration calorimeter (Malvern Instruments) at 37 °C, the reaction cell containing 50 μM YiaC (or CobB) was titrated with 500 μM different actyl-CoA (or actylated peptides). The volume of the first injection was 0.5 μl of 500 μM actyl-CoA (or actylated peptides), and the following 18 injections were 2.0 μl. The binding isotherm was fit with the Origin 8.0 software (OriginLab). Each group had three biological repetitions.

### Nano-HPLC assay for determination of the reacted peptides of YiaC and CobB

CobB reaction was described as previously^8^. In detail, lactylated or acetylated peptides (50 μM) with 1 μM CobB were incubated in CobB reaction buffer [50 mM Tris-HCl, 137 mM NaCl, 2.7 mM KCl, 1 mM MgCl_2_, 1 mM DTT, and 1 mM NAD^+^ (pH 8.5)] with or without 10 mM NAM for 2 h at 37 °C. For YiaC reaction, unmodified peptide (50 μM) with 5 μM YiaC were incubated in YiaC eaction buffer [100 mM HEPES, 100 mM MgCl_2_, 10 mM KCl, and 0.1 mM lactyl-CoA or acetyl-CoA (PH = 7)] for 2 h at 37 °C. After quenching reaction with equal volume of 10% (v/v) trifluoroacetic acid, the samples were spun for 10 min at 18,000 × *g* to separate the enzyme. Samples were separated by a 28-min HPLC gradient [linear gradient from 5 to 50% HPLC buffer B (0.1% formic acid in acetonitrile) for 3 min and then to 100% buffer B for 20 min and keeping 100% buffer B for 5 min].

### Determination of *k*_*cat*_ and *K*_*m*_ of YiaC and CobB

This method was described previously^8^. CobB was incubated with modified peptides (AETAEK(la)YGDEQVK or AETAEK(ac)YGDEQVK, from GpmA) in the CobB reaction buffer at 37 °C for 2 min. Meanwhile, YiaC was incubated with unmodified peptide (TICHDKAFVK, from PncB) in the YiaC reaction buffer. The concentrations of the peptides were 5, 10, 16, 32, 60, and 285 uM, and reaction time were 1, 5, 15, and 30 min. After quenching the reaction by adding equal volume of 10% (v/v) trifluoroacetic acid and cleaning with C18 ZipTips, the products were analyzed by Autoflex III TOF/TOF mass spectrometer (Bruker Daltonics) with a reflex positive-ion mode equipping delayed ion extraction. 0.1 μl samples mixed with 2,5-dihydroxybenzoic acid (DHB) were analyzed by MS. An acceleration voltage of 20 kV was used. MS data were analyzed using FlexAnalysis software for spectral processing and peak detection. The enzyme kinetics were analyzed by Prism GraphPad 8.0 software.

### SILAC labeling and sample preparation

As described previously^8^, *E. coli* MG1655, *E. coli* MG1655 Δ*yiaC* and Δ*cobB* were cultured at 37 °C in M9 minimal medium with 0.2% glucose and L-lysine (100 mg ml^–1^) or ^13^C_6_-lysine (100 mg ml^–1^) shown as Fig. 3A. Cells were harvested during the mid-exponential phase then sonicated on ice in PBS. After centrifugation (20,000 g) at 4 °C for 20 min, the supernatant was collected. Equal amounts of proteins from *E. coli* MG1655 and MG1655 Δ*yiaC* (or Δ*cobB*) were mixed and precipitated by trichloroacetic acid, precipitates were dissolved in 100 mM NH4HCO3 with overnight digestion by trypsin (trypsin: protein ratio, 1:50). Digested products were incubated with 10 mM DTT at 37 °C for 1 h, followed by alkylation with 20 mM iodoacetamide for 45 min at room temperature under darkness. Excess iodoacetamide was blocked with 20 mM cysteine, a last digestion was performed at a ratio of 1:100 for 4 h. Products were desalted by SepPak C18 cartridges (Waters) and dried.

### Immunoaffinity enrichment

Tryptic peptides were redissolved in NETN buffer (50 mM Tris-HCl pH 8.0, 100 mM NaCl, 1 mM EDTA, 0.5% Nonidet P-40) and incubated with anti-lactyllysine antibody-conjugated protein A agarose beads (PTM Biolabs) at 4 °C overnight, with gentle rotation. The incubated beads were washed three times with NETN buffer, twice with ETN buffer (50 mM Tris-HCl pH 8.0, 100 mM NaCl and 1 mM EDTA) and three times with water. Bound peptides were eluted three times with 1% trifluoroacetic acid. Finally, the eluates were dried and cleaned with C18 ZipTips (Millipore Corp) before nano-HPLC–MS/MS analysis.

### HPLC-MS/MS analysis for Kla

Enriched peptides were analyzed by HPLC-MS/MS. Samples was reconstituted in 0.1% formic acid and then injected into a nano-LC system (EASY-nLC 1200, Thermo Fisher Scientific). Peptides were resolved by a 75 µm inner diameter, 25 cm-long C18 column (3 µm, Dr. Maisch GmbH, Ammerbuch, Germany) at flow rate of 300 nL/min. Gradient elution was performed with 2-45% HPLC buffer B (0.1% formic acid in 80% acetonitrile) for 90 min then transition to 100% buffer B for 15 min, and keep with 100% buffer B for 5 min. An Orbitrap Eclipse Tribrid mass spectrometer with a FAIMS Pro Interface (Thermo Fisher Scientific) was employed for MS analysis. Spray voltage was set to 2.1 kV, funnel RF level at 40, and ion transfer tube temperature at 320 °C. Mass spectrometric analysis was carried out in data-dependent acquisition (DDA) mode of the most intense precursors, and data were collected by Xcalibur (v.4.0.27.19). Combination of −40V/−60V/−80V FAIMS CVs (compensation voltage) were set to run DDA mode for 1 s cycle to build a big cycle of 3 s. The orbitrap mass analyzer was used as the MS1 detector with 60,000 resolution and scan range 350–1600 m/z. The normalized AGC target and maximum injection time were set at 100% /20 ms for MS1, and 200% /30 ms for MS2. The orbitrap mass analyzer was used as the MS2 detector with 17,500 resolution. Precursor ions with charges of +2 to +5 were isolated for MS2, and dynamic exclusion time was set at 50 s. The MS2 isolation window was 1.6 Da, and a normalized HCD (higher-energy collision-induced dissociation) collision energy of 30% was used for precursor fragmentation.

### Database search and data filter criteria for Kla

The database search and filter criteria were performed as described previously^8^. In detail, raw data were searched by MaxQuant (v.1.5.5.1) with UniProt *E. coli* K12 protein database (Proteome ID: UP000474145) and an overall false discovery rate for peptides of less than 1%. Peptide sequences searching was set as trypsin specificity, two missed cleavages for maximum and seven for minimal peptide length. Carbamidomethylation on Cys was specified as fixed modification. Lactylation on lysine, oxidation of methionine and acetylation on the peptide N terminus were fixed as variable modifications. Mass tolerances were set at ±10 ppm for precursor ions and ±0.02 Da for MS/MS. Lactylated peptides with a score of <40 and localization probability of <0.75 were further excluded. We normalized all the Kla peptide ratios by the ratios of the corresponding protein levels.

### GO analysis

The GO (Gene Ontology) function in three categories BPs (Biological Processes), CC (Cellular Component), and MF (molecular functions) pathway enrichment analyzed through the R/Bioconductor package “clusterProfiler” (version:4.0)^67^. The p-value cutoff = 0.05 and q-value cutoff = 0.2 were selected as the cutoff criteria. Statistical significance was calculated with a two-sided unpaired fisher’s exact test. Benjamini and Hochberg correction was used to adjust *P*-values.

### Lactylation and delactylation reaction by YiaC and CobB

The purified recombinant YiaC (15 μM) and candidate substrate proteins (GltA, PncB, 20 μM) were incubated in YiaC reaction buffer at 25 °C for 10 h. The purified recombinant CobB (15 μM) and candidate substrates (GpmA, Mdh, PykF, 20 μM) were incubated in CobB reaction buffer at 25 °C for 10 h. The reaction products were analyzed by Western blotting with anti-lactylation.

### In vitro assay for PncB activity

The activity of purified PncB, PncB^K381Q^, and PncB^K381R^ were measured as described^68^. In detail, the purified proteins were added in the reaction buffer [50 mM Tris-HCl, 10 mM MgCl_2_, 2.5 mM dithiothreitol, 1 mM ATP, 25 g of bovine serum albumin, 1 mM nicotinic acid and 1 mM 5-phosphoribosyl-1-pyrophosphate (pH 7.5)] at 37 °C for 2 h. The reactions were measured by HPLC-MS/MS to detect the production of nicotinic acid. Separation was performed on a Vanquish UHPLC system (Thermo Fisher Scientific) with an ACQUITY UPLC BEH C18 column (100 × 2.1 mm, 1.7 μm). Mobile phase A was 0.1% formic acid in water. Mobile phase B was 0.1% formic acid in methanol. Gradient elution (0.0 min, 0% B; 2 min, 15% B; 4 min, 100% B; 5 min, 0% B) was carried out at a flow rate of 0.2 ml/min and a constant column temperature of 20 °C. Injection volume was 2 μl. Nicotinic acid detection was performed on an Orbitrap Exploris 480 spectrometer (Thermo Fisher Scientific, Bremen, Germany) operated with ESI probe. Data were acquired under the following parameters: sheath gas ((N2), 35 Arb; aux gas (N2), 10 Arb; spray voltage, 3500 V (positive mode); and in transfer tube temperature, 320 °C. An SIM was applied for data acquisition.

### In vitro assay for GpmA activity

The activity of purified GpmA, GpmA^K106Q^ and GpmA^K106R^ were measured as described^69^. In detail, the purified recombinant GpmA, GpmA^K106Q^ or GpmA^K106R^ were incubated in reaction buffer [100 mM Tris-HCl, 100 mM KCl, 5 mM MgCl_2_, 1 mM ADP, and 0.2 mM NADH (PH 7.0)] with 0.5 U enolase, 0.5 U pyruvate kinase, 0.1 U Ldh, and 2 mM 3- phosphoglycerate for 1 h at 30 °C, and samples without GpmA served as a blank. The oxidation of NADH was measured as GpmA activity by autofluorescence in 340 nm.

### In vitro assay for PykF activity

The activity of purified PykF, PykF^K382Q^ and PykF^K382R^ were measured as described^70^. In detail, the purified recombinant PykF, PykF^K382Q^, and PykF^K382R^ were incubated in reaction buffer [50 mM Tris-HCl, 200 mM KCl, 15 mM MgCl_2_, 25% glycerol, 175 μM NADH, 5 mM phosphoenolpyruvate, 5 mM ADP, and 5 mM fructose 1,6-bisphosphate (PH 8.0)] with 1 U lactate dehydrogenase for 10 min at 32 °C, and samples without PykF served as a blank. The oxidation of NADH was measured as PykF activity by autofluorescence in 340 nm.

### Intracellular assay for PykF activity

*E. coli* in 5 ml culture medium were harvested. The assay was performed with the Pyruvate Kinase Activity Assay Kit (Sigma–Aldrich, MAK072) following the protocol from manufacturer.

### Drawing growth curve

As previous described^8^, the strains were cultured in LB medium overnight, then diluted 1:1000 in LB or M9 medium (with 1% glucose or 1% pyruvate) and incubated at 37 °C with 96-well plates, and each strain was added to three wells. The absorbance at 600 nm at initiation was set as a blank and measured every hour using a spectrophotometer. All the growth dynamic curves were drawn using Origin 8.0.

### Molecular dynamics simulation

We performed two systems PykF/PykF K382la-FBP for 100 ns Molecular dynamics (MD) simulations for three times. The initial coordinates of the systems were obtained from X-ray crystal structures with PDB ID 1PKY. All MD simulations were performed using GROMACS 2019.6 (set defaults values as parameters). The software package with Gromos 54A7 force field was applied to describe the PykF, and Gromos 54A8 force field was used for the PykF K382la. Gromos 54A7 and Gromos 54A8 were downloaded from Vienna-PTM. The ligand FBP force fields were obtained from the Automated Topology Builder (ATB) (Version 3.0) database (https://atb.uq.edu.au/molecule.py?molid=1134439#panel-md). The complex systems were analyzed by MD simulations in a cubic box with the SPC water model. To neutralize the systems, chloride and sodium ions were randomly added to the simulation box. During the MD simulations, the long-range coulombic interactions were handled using the particle mesh Ewald (PME) method. The energy minimization of the whole system was carried out for 50,000 steps with the steepest descent method. Subsequently, 500 ps of NVT (Berendsen temperature coupled with constant particle number, volume, and temperature) and 500 ps of NPT (Parrinello–Rahman pressure coupled with constant particle number, pressure, and temperature) were performed to maintain the stability of the system (300 K, 1 bar). After stabilizing all thermodynamic properties, the molecular system was simulated for 100 ns with a time interval of 2 fs, whereas the coordinates for all models were stored every 2 ps. Root mean square deviations (RMSD) and distances between the nitrogen atom of K382 side chain and the central carbon atom of FBP were evaluated using the analysis tools within GROMACS 2019.6. All visualization is done through PyMOL and Xmgrace 2.3.7 software. Codes files, input parameters files and initial and final configurations files related to two MD simulations systems (PykF-FBP and PykF K382la-FBP) are provided as a MD simulation Source Data.

## Supplementary information

Supplementary information

Description of Additional Supplementary files

Supplementary Data 1

Supplementary Data 2

Supplementary Data 3

Supplementary Data 4

Supplementary Data 5

Supplementary Data 6

Supplementary Data 7

## Data Availability

All data needed to evaluate the conclusions in the paper are present in the paper and/or the Supplementary Materials. Additional data related to this paper may be requested from the authors. The MS proteomics data have been deposited to the ProteomeXchange Consortium (http://proteomecentral.proteomexchange.org) via the iProX partner repository with the dataset identifier PXD030345 and PXD030346. The ligand FBP force fields were obtained from the Automated Topology Builder (ATB) (Version 3.0) database (https://atb.uq.edu.au/molecule.py?molid=1134439#panel-md). The following crystal structure was used for analysis PDB ID 1PKY. Codes files, input parameters files, and initial and final configurations files related to two MD simulations systems (PykF-FBP and PykF K382la-FBP) are provided as a MD simulation Source Data. Source data are provided with this paper.

## Source data

Source Data
