# Supplementary material for: YiaC and CobB regulate lysine lactylation in Escherichia coli
Source: Nat Commun. 2022 Nov 4;13:6628. doi: 10.1038/s41467-022-34399-y (PMC9636275; doi:10.1038/s41467-022-34399-y)
Supplement: Supplementary file 1 — Supplementary information [file 41467_2022_34399_MOESM1_ESM.pdf]

Supplementary Information

YiaC and CobB regulate lysine lactylation in *Escherichia coli*

Hanyang Dong[1,2,#], Jianji Zhang[1,#], Hui Zhang[1], Yue Han[1], Congcong Lu[3], Chen Chen[1], Xiaoxia Tan[1], Siyu Wang[1], Xue Bai[1], Guijin Zhai[1], Shanshan Tian[1], Tao Zhang[1], Zhongyi Cheng[4], Enmin Li[2,5], Liyan Xu[2,*] and Kai Zhang[1,6,7,*]

[1] The Province and Ministry Co-sponsored Collaborative Innovation Center for Medical Epigenetics, Key Laboratory of Immune Microenvironment and Disease (Ministry of Education), Tianjin Key Laboratory of Medical Epigenetics, Department of Biochemistry and Molecular Biology, School of Basic Medical Sciences, Tianjin Medical University, Tianjin 300070, China

[2] Guangdong Provincial Key Laboratory of Infectious Diseases and Molecular Immunopathology, Institute of Oncologic Pathology, Shantou University Medical College, Shantou 515041, Guangdong, China

[3] College of Life Sciences, Nankai University, Tianjin 300071, China

[4] Jingjie PTM Biolab (Hangzhou) Co. Ltd, Hangzhou 310018, Zhejiang, China

[5] The Key Laboratory of Molecular Biology for High Cancer Incidence Coastal Chaoshan Area, Department of Biochemistry and Molecular Biology, Shantou University Medical College, Shantou 515041, Guangdong, China

[6] Tianjin Key Laboratory of Retinal Functions and Diseases, Eye Institute and School of Optometry, Tianjin Medical University Eye Hospital, Tianjin Medical University, Tianjin 300070, China

[7] Tianjin Key Laboratory of Digestive Diseases, Department of Gastroenterology and Hepatology, Medical University General Hospital, Tianjin Medical University, Tianjin 300070, China

[#] These authors contributed equally to this work.

***** Corresponding author: kzhang@tmu.edu.cn (K.Z.) and lyxu@stu.edu.cn (L.X.)

Supplementary Figures

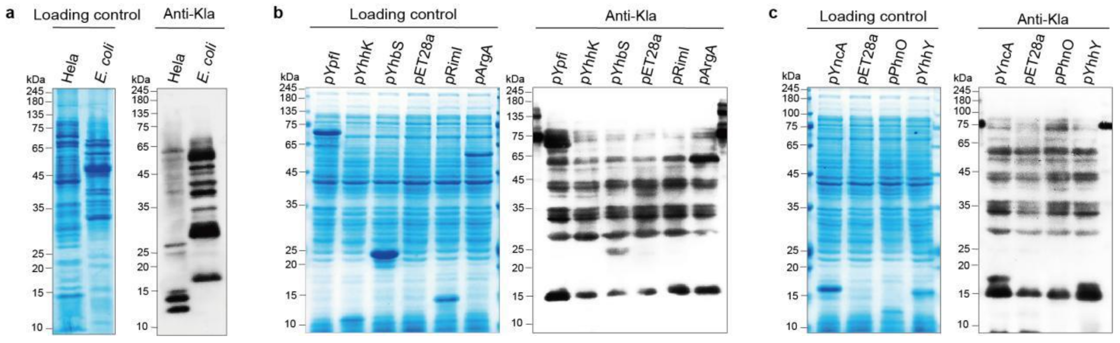

Supplementary Fig. 1. The Kla levels of whole cell lysates were analyzed by western blotting. **a** Western blotting detection the Kla level of Hela and *E. coli* MG1655 for whole cell lysates. **b** and **c** Western blotting detection the Kla level of *E. coli* for whole cell lysates, in which *E. coli* BL21 (λDE3) were transferred with empty vector (pET28a) as control and GNAT *genes*-pET28a vectors (pGNAT). All immunoblots had three biological repetitions, with similar results. All strains were cultured in LB medium at 37 °C.

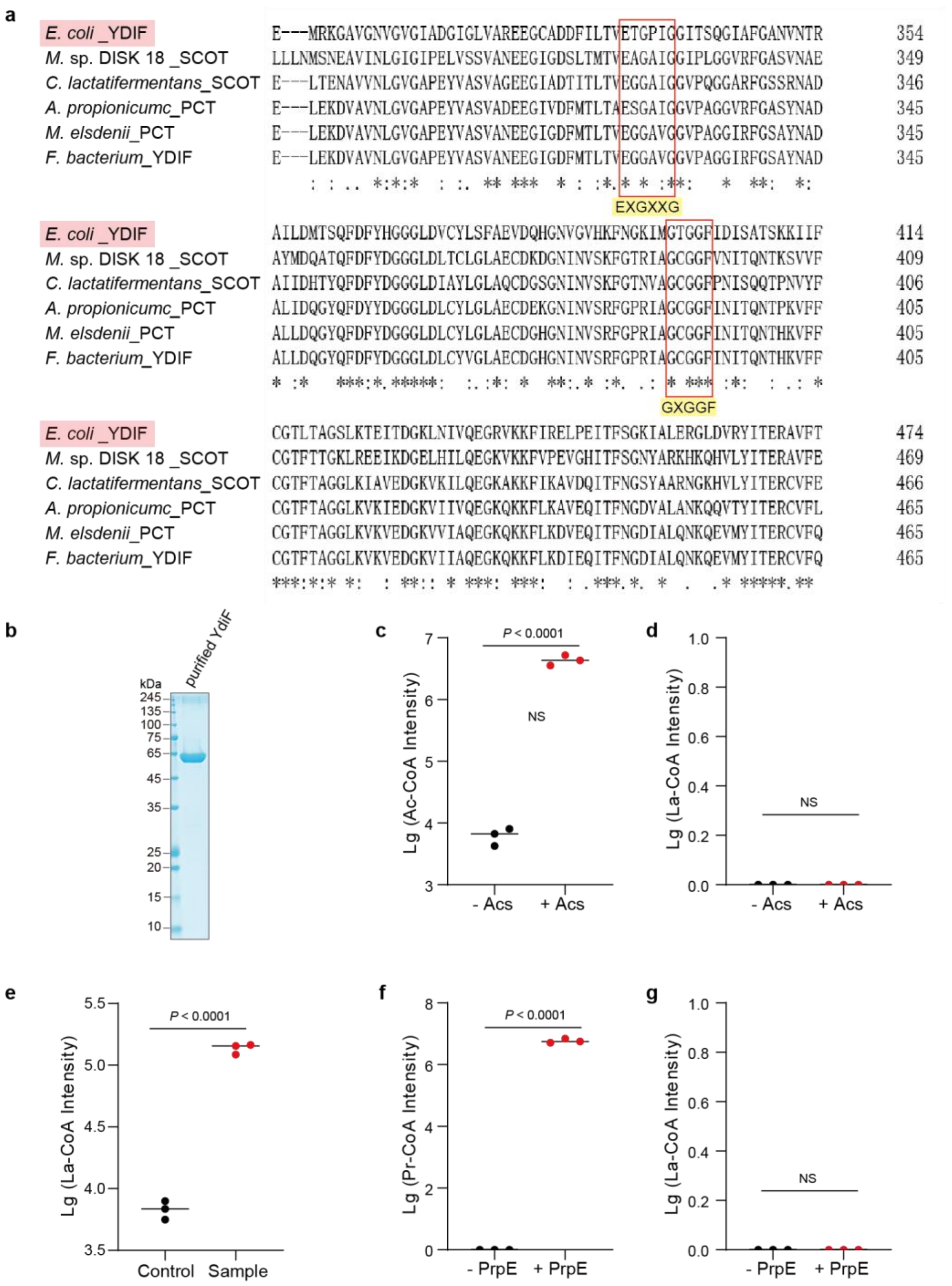

Supplementary Fig. 2. Lactyl-CoA transferase existing in *E. coli*. a Amino acid sequence alignment of six CoA-transferases. The conserved sequence motifs EXGXXG and GXGGF are marked by boxes. **b** SDS-PAGE analysis of purified YdiF. **c** is performed produce acetyl-CoA (Ac-CoA) by Acs. – Acs: incubation acetate with coenzyme A (HSCoA), + Acs: incubation acetate and HSCoA with Acs (n=3 biological repeats). **d** is performed produce lactyl-CoA (La-CoA) by Acs. – Acs: incubation lactate with HSCoA, + Acs: incubation lactate and HSCoA with Acs (n=3 biological repeats). **e** Whole-cell lysate catalyzes lactate to La-CoA. LC-MS/MS detection La-CoA of whole cell lysate as control, lactate and HSCoA incubation with whole cell lysate as sample (n=3 biological repeats). **f** is performed produce propionate CoA (Pr-CoA) by

PrpE. – PrpE: incubation propionate with HSCoA, + PrpE: incubation propionate and HSCoA with PrpE (n=3 biological repeats). **g** is performed produce La-CoA by PrpE. – PrpE: incubation lactate with HSCoA, + PrpE: incubation lactate and HSCoA with PrpE (n=3 biological repeats). Data are presented as mean values ± SEM, two-tailed Student's t test, NS means not significant. P-values are indicated in the figure.. Source data are provided as a Source Data file.

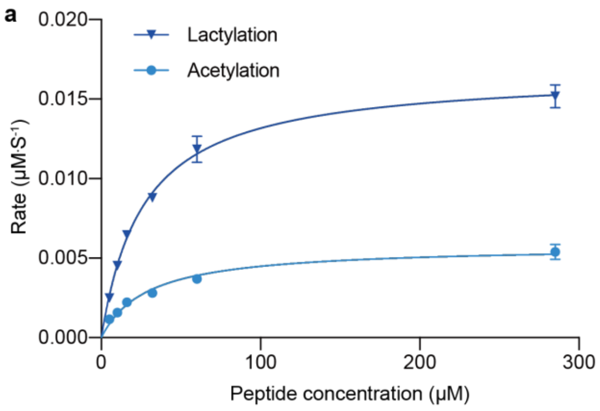

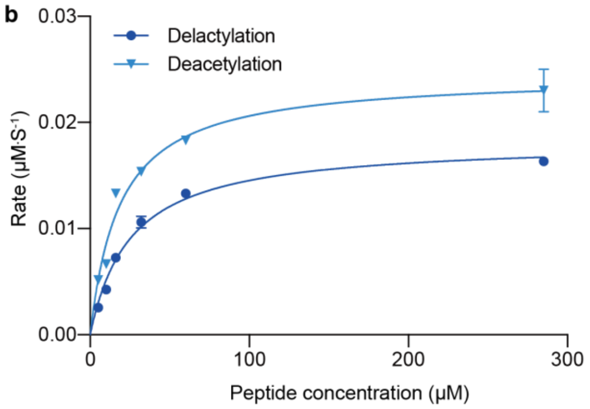

Supplementary Fig. 3. Kinetic curves of enzymatic reaction. a Drawing the kinetic curves of YiaC by Prism GraphPad 8 (n=3 biological repeats). **b** Drawing the kinetic curves of CobB by Prism GraphPad 8 (n=3 biological repeats). Data are presented as mean values ± SEM, two-tailed Student's t test. Source data are provided as a Source Data file.

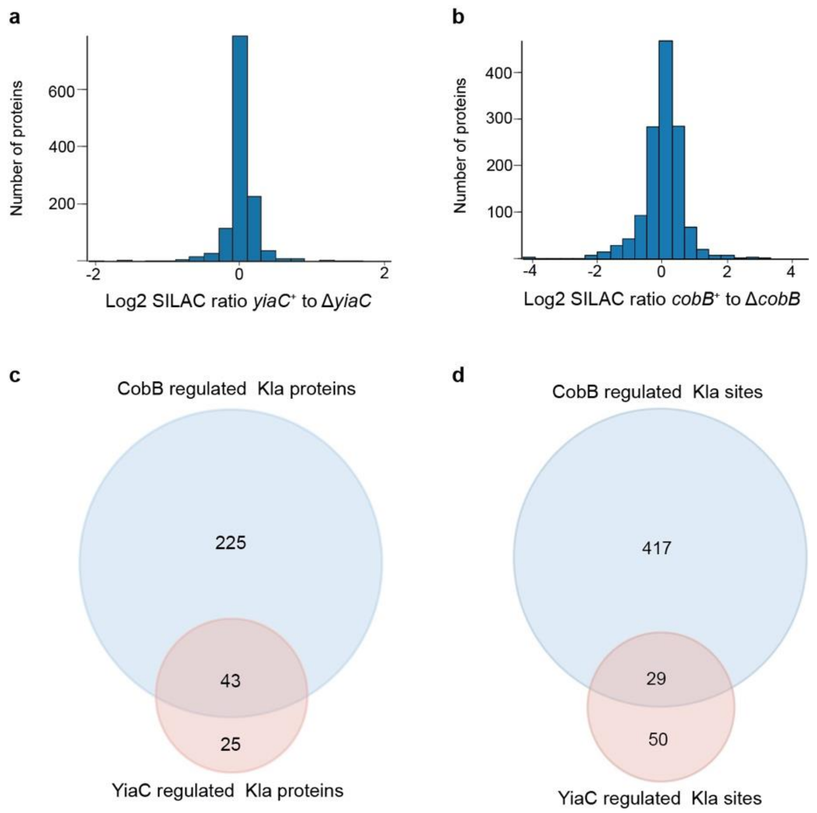

Supplementary Fig. 4. Histogram showing relative protein abundance. a Histogram showing relative protein abundance between *E. coli* MG1655 *yiaC*$^{+}$ and Δ*yiaC*. **b** Histogram showing relative protein abundance between *E. coli* MG1655 *cobB*$^{+}$ and Δ*cobB*. **c** Comparation of endogenous substrates between YiaC and CobB. **d** Comparison of endogenous substrate sites between YiaC and CobB.

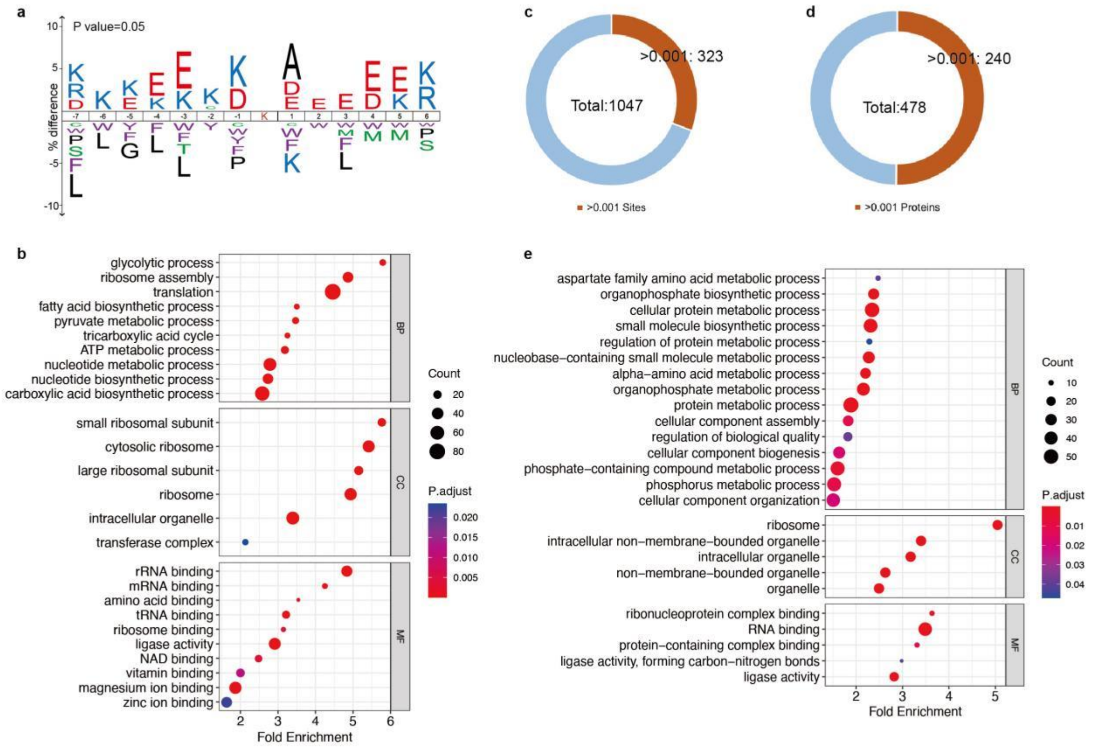

Supplementary Fig. 5. Characterization of the Kla proteome in *E. coli*. a Sequence motif logo shows a representative sequence for all Kla sites. **b** GO analysis for all Kla proteins of proteome. **c** The proportion of Kla sites with high frequency. **d** The proportion of proteins with high frequency Kla. **e** GO analysis for proteins with high frequency Kla. The p-value cutoff = 0.05 and q-value cutoff = 0.2 were selected as the cutoff criteria. Benjamini & Hochberg correction was used to adjust P values.

a

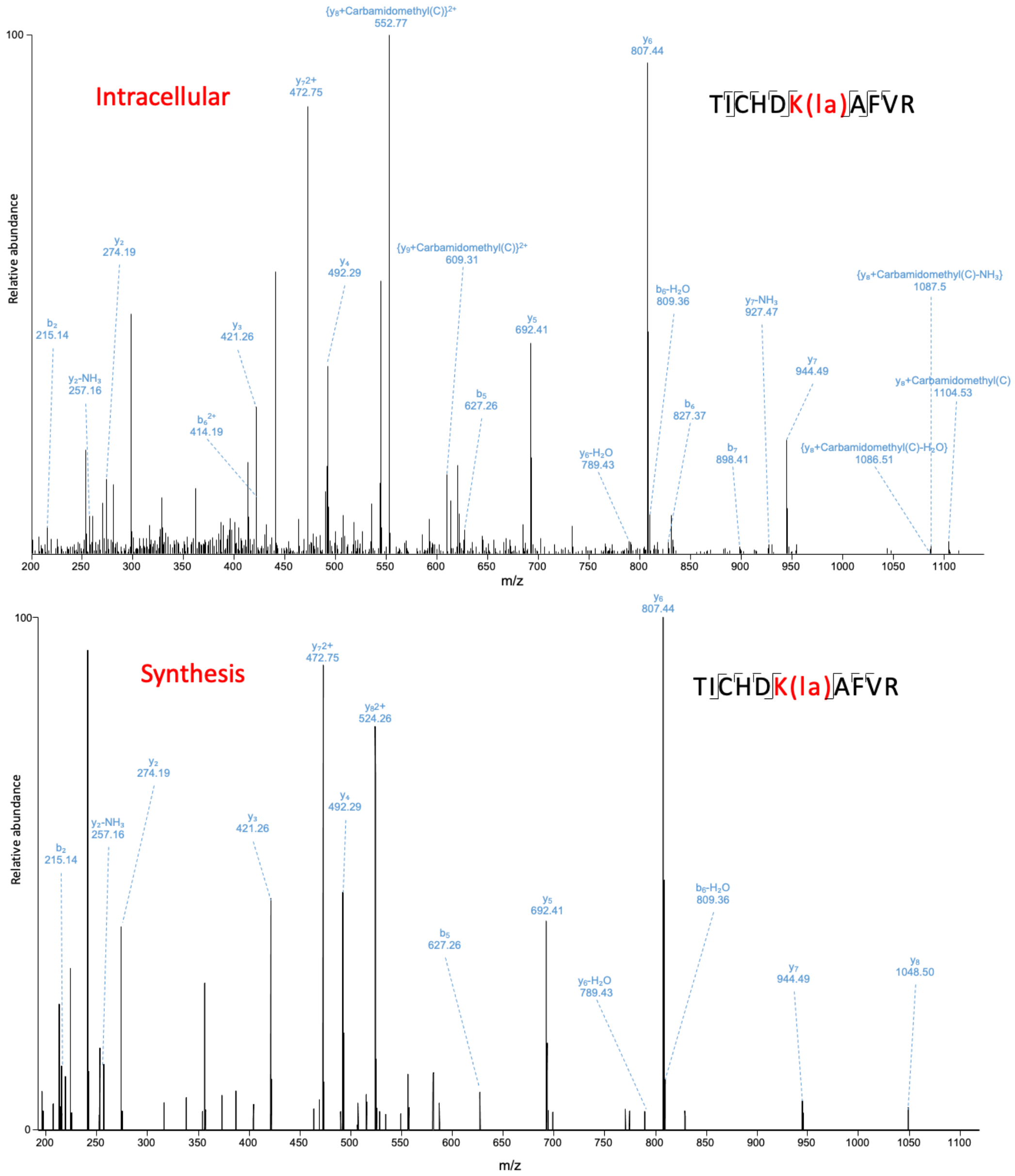

Intracellular
TICHDK(la)AFVR
Relative abundance
100
m/z
{y8+Carbamidomethyl(C)}2+
552.77
y6
807.44
y72+
472.75
y2
274.19
y4
492.29
{y9+Carbamidomethyl(C)}2+
609.31
b2
215.14
y3
421.26
y2-NH3
257.16
b62+
414.19
b5
627.26
y5
692.41
y6-H2O
789.43
b6-H2O
809.36
b6
827.37
b7
898.41
y7-NH3
927.47
y7
944.49
{y8+Carbamidomethyl(C)-NH3}
1087.5
y8+Carbamidomethyl(C)
1104.53
{y8+Carbamidomethyl(C)-H2O}
1086.51
Synthesis
TICHDK(la)AFVR
Relative abundance
100
0
m/z
y6
807.44
y72+
472.75
y82+
524.26
y2
274.19
y4
492.29
y2-NH3
257.16
y3
421.26
b2
215.14
b6-H2O
809.36
y5
692.41
b5
627.26
y6-H2O
789.43
y7
944.49
y8
1048.50

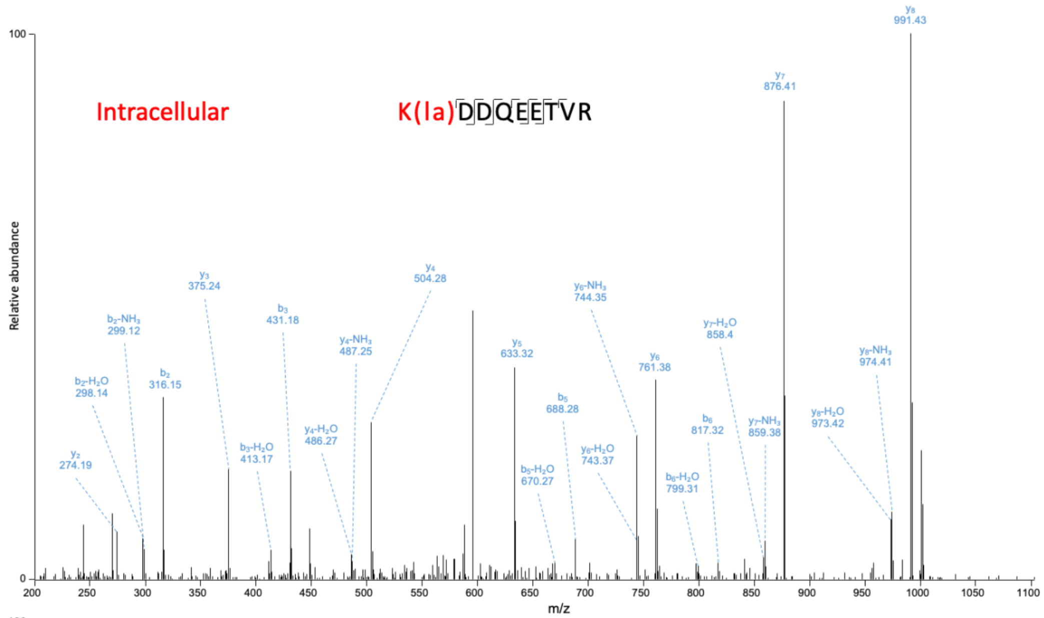
Intracellular
K(la)DDQEETVR
Relative abundance
m/z
y2 274.19
b2-H2O 298.14
b2-NH3 299.12
b2 316.15
y3 375.24
b3-H2O 413.17
b3 431.18
y4-H2O 486.27
y4-NH3 487.25
y4 504.28
y5 633.32
b5-H2O 670.27
b5 688.28
y6-H2O 743.37
y6-NH3 744.35
y6 761.38
b6-H2O 799.31
b6 817.32
y7-H2O 858.4
y7-NH3 859.38
y7 876.41
y8-H2O 973.42
y8-NH3 974.41
y8 991.43

b

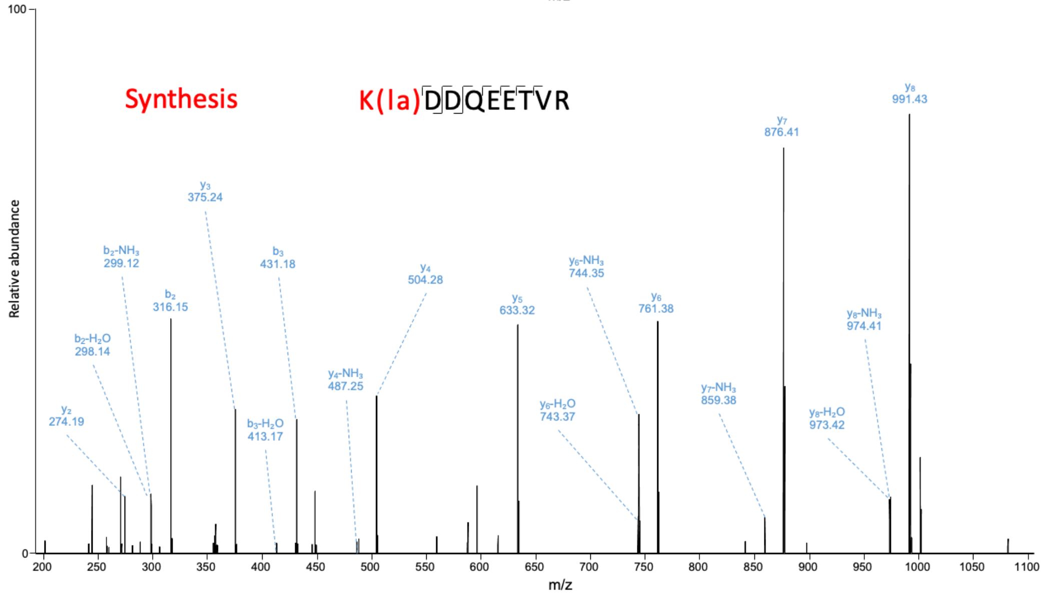
Synthesis
K(la)DDQEETVR
Relative abundance
m/z
y2 274.19
b2-H2O 298.14
b2-NH3 299.12
b2 316.15
y3 375.24
b3-H2O 413.17
b3 431.18
y4-NH3 487.25
y4 504.28
y5 633.32
y6-H2O 743.37
y6-NH3 744.35
y6 761.38
y7-NH3 859.38
y7 876.41
y8-H2O 973.42
y8-NH3 974.41
y8 991.43

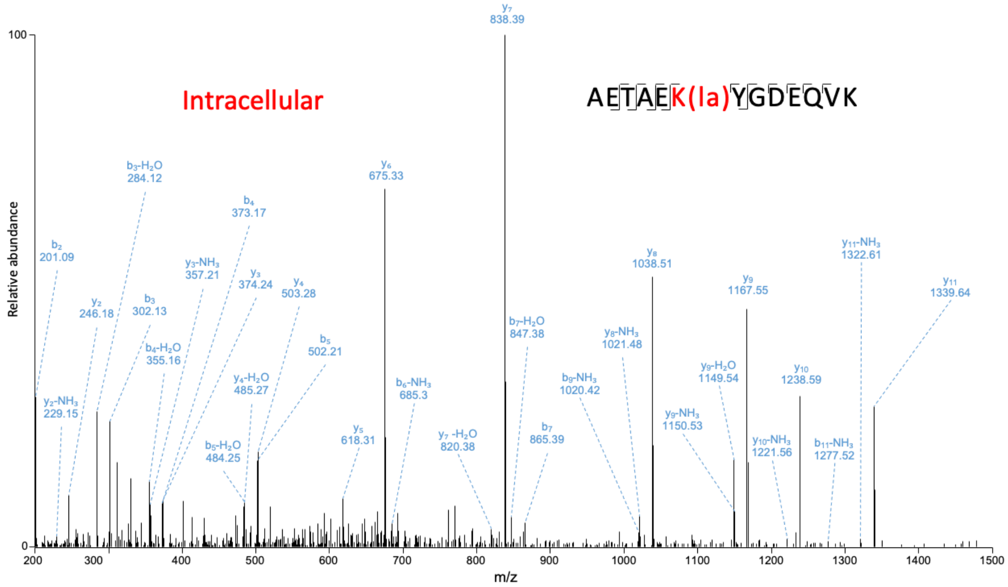

c

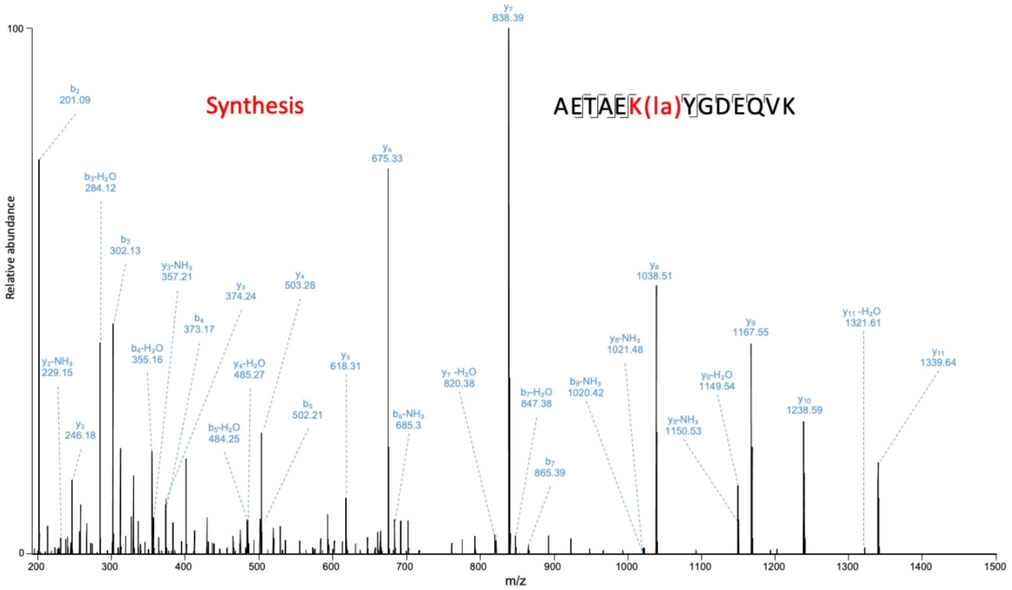

Supplementary Fig. 6. The MS/MS spectra of identified Kla peptides and its counterpart synthetic peptides. a The MS/MS spectra of peptide (TICHDK(la)AFVR, PncB) from proteome of Δ*yiaC*. **b** The MS/MS spectra of peptide (K(la)DDQEETVR,Kad) from both proteome of Δ*yiaC* and Δ*cobB*. **c** The MS/MS spectra of peptide (AETAEK(la)YGDEQVK, GpmA) from both proteome of Δ*yiaC* and Δ*cobB*.

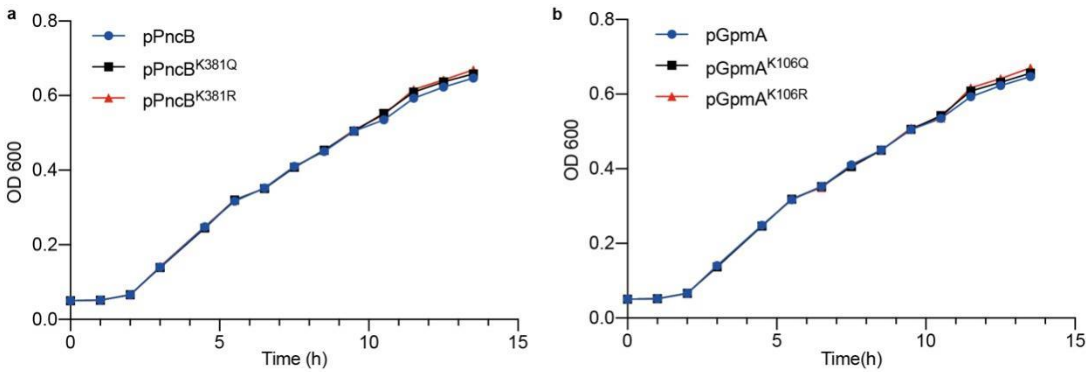

Supplementary Fig. 7. Kla on PncB and GpmA have no effect on growth of *E. coli* MG1655 in LB medium. a Measurement growth curve of PncB-overexpressing *E. coli* MG1655 (pPncB, pPncB^{K381Q} and pPncB^{K381R}) cultured in LB medium at 37°C with 96-well plates, from three independent assays. OD600, optical density at 600 nm. **b** Measurement growth curve of GpmA-overexpressing *E. coli* MG1655 (pGpmA, pGpmA^{K106Q} and pGpmA^{K106R}) cultured in LB medium at 37°C with 96-well plates, from three independent assays. OD600, optical density at 600 nm. Data are presented as mean values ± SEM, two-tailed Student's t test. Source data are provided as a Source Data file.

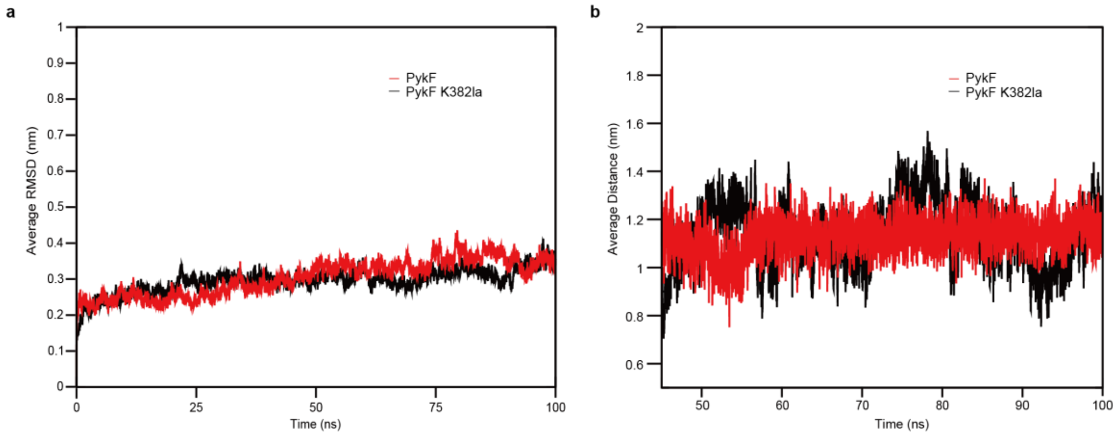

Supplementary Fig. 8. The RMSDs and distances of the molecular dynamics (MD) simulations. a The average RMSDs of three molecular dynamics (MD) simulations for PykF-FBP (red) and PykF K382la-FBP (black), respectively. **b** The average distances of three molecular dynamics (MD) simulations from 45 ns to 100 ns for PykF-FBP (red) and PykF K382la-FBP (black), respectively. The average distances indicate the distance between the PykF K382 side chain nitrogen atom and the FBP central carbon atom. Source data are provided as a Source Data file.
