# Supplementary material for: YiaC and CobB regulate lysine lactylation in Escherichia coli
Source: Nat Commun. 2022 Nov 4;13:6628. doi: 10.1038/s41467-022-34399-y (PMC9636275; doi:10.1038/s41467-022-34399-y)
Supplement: Supplementary file 2 — Description of Additional Supplementary files [file 41467_2022_34399_MOESM2_ESM.docx]

**Supplementary Information**

**Supplementary Datas**

File Name: **Supplementary Data 1.**

Description: The data of ITC experiments.

File Name: **Supplementary Data 2.**

Description: The pertinent data of enzyme kinetics.

File Name: **Supplementary Data 3.**

Description: The quantification result of lactylated proteomes with *E. coli* MG1655 **Δ*y****iaC* and *yiaC^+^*.

File Name: **Supplementary Data 4.**

Description: The quantification result of lactylated proteomes with *E. coli* MG1655 Δ*cobB* and *cobB^+^*.

File Name: **Supplementary Data 5.**

Description: The lactylated proteomes of *E. coli*.

File Name: **Supplementary Data 6.**

Description: The Kla intensity corrected by protein intensity.

File Name: **Supplementary Data 7.**

Description: The list of reagents, bacterial strains, and plasmids used in this paper.

Supplementary Data 1 to 7 are available in the Supplementary Data files.
